# Novel innovative computer-based test (Inno-CBT) item types for national licensing examinations for health care professionals

**DOI:** 10.1186/s12909-023-04444-5

**Published:** 2023-08-09

**Authors:** Kwang-Hoon Chun, Hye Kyung Jin, Jeong-Hyun Yoon, Myeong Gyu Kim, Kyung Hee Choi, Eunyoung Kim, Hyunah Kim, Jin-Ki Kim, Gyudong Kim, Kyungim Kim, Ju-Yeun Lee, Eun Kyoung Chung, Young Sook Lee, Sandy Jeong Rhie

**Affiliations:** 1 Gachon Institute of Pharmaceutical Sciences, College of Pharmacy, Gachon University, Incheon, South Korea; 2 College of Pharmacy, Dongguk University-Seoul, Goyang, South Korea; 3 College of Pharmacy, Pusan National University, Busan, South Korea; 4 College of Pharmacy and Graduate School of Pharmaceutical Sciences, Ewha Womans University, Seoul, South Korea; 5 College of Pharmacy, Gachon University, Incheon, South Korea; 6 Department of Health, Social and Clinical Pharmacy, College of Pharmacy, Chung-Ang University, Seoul, South Korea; 7 College of Pharmacy, Sookmyung Women’s University, Seoul, South Korea; 8 College of Pharmacy, Institute of Pharmaceutical Science and Technology, Hanyang University, Ansan, South Korea; 9 College of Pharmacy, Chonnam National University, Gwangju, South Korea; 10 College of Pharmacy, Korea University, Sejong, South Korea; 11 College of Pharmacy and Research Institute of Pharmaceutical Sciences, Seoul National University, Seoul, South Korea; 12 Department of Pharmacy, College of Pharmacy, Kyung Hee University, Seoul, South Korea; 13 College of Pharmacy, Keimyung University, Daegu, South Korea

**Keywords:** Computer-based test, Innovative CBT item types, Examination, Health Personnel, Licensure

## Abstract

**Background:**

An effective test mechanism to evaluate clinical knowledge and skills of the entry-level healthcare professionals is important for providing clinical competency and improving patient care. This study aimed to develop novel, innovative computer-based test (Inno-CBT) item types for application in the national examination of Korean healthcare professionals.

**Methods:**

This exploratory study was conducted from May 2021 to March 2022 by a team of faculty members from pharmacy schools in South Korea. A literature search using PubMed, Google Scholar, RISS, Web of Science, and KoreaMed was performed. Forum presentations, media articles, and previous reports by the Korea Health Personnel Licensing Examination Institute (KHPLEI) were included. Workshops were held, information and ideas were collected and conceptualized, and item types were designed, drafted, and refined. By repeating this process, the Inno-CBT item types were finalized.

**Results:**

Forty-one Inno-CBT item types with 28 subtypes were developed. New digital technologies, such as a reactive responsive media interface, an animation insertion, multimedia embedding, and network surfing, were utilized in these novel types. It was anticipated that these Inno-CBT item types would effectively measure abilities in healthcare knowledge, problem-solving skills, and professional behaviors. Some potential barriers to implementing the Inno-CBT item types include item difficulty, operational unfamiliarity, complexity in scoring protocols, and network security.

**Conclusions:**

A variety of styles of novel Inno-CBT item types were developed to evaluate the multifaceted and in-depth professional abilities required for healthcare professionals. Prior to implementing these item types in the national examination, item validation and technical support should be conducted.

**Supplementary Information:**

The online version contains supplementary material available at 10.1186/s12909-023-04444-5.

## Introduction

The importance of clinical competency in early-stage professionals has been emphasized during the last decade. Effort has been made to improve the school curriculum of experiential learning programs by offering different healthcare environments and advanced health technologies [1]. At the same time, an effective test mechanism has been requested to evaluate candidates’ clinical knowledge and skills to ensure their readiness for entering the healthcare profession.

A computer-based test (CBT) integrating information technology (IT) has been introduced to licensure tests [2]. Compared to a paper-based test (PBT), a CBT offers several advantages, including multiple assessment methods, short test times, and more reliable assessment approaches [3, 4]. A CBT also provides longitudinal data from assessments, allowing trends and improvements in learning performance to be identified. In addition, CBT can easily tailor test questions based on difficulty level and assessment purpose [5]. Moreover, examinees can obtain evaluation results promptly from an automated scoring protocol [3–6]. Nonetheless, the currently used CBT item types generally have too simple structures to assess the precise skills and complex concepts of health sciences often encountered in current practice environments. The item types of the examination managed by the Korea Health Personnel Licensing Examination Institute (KHPLEI) are predominantly composed of multiple choice item types (MCITs) of A-type (one single best answer among five options). Meanwhile, new CBT item types have been requested by the National Council of State Boards of Nursing [7]; however, the item types newly developed are limited in diversity, and their applicability has not been fully established yet.

In this study, we introduce novel, innovative CBT (Inno-CBT) item types, which incorporate the functions of the diverse user interface of computer-based technology. This study aimed to improve the test quality and their usability in the national examinations for healthcare professionals.

## Methods

### Data collection and identification of CBT item types

This was an exploratory study conducted from May 2021 to March 2022 and involved 14 faculty members from various pharmacy schools in South Korea. These researchers were specifically chosen due to their previous experience in developing test questions for the Korea Health Personnel Licensing Examination (KHPLE) and held Ph.D. degrees in fields such as basic sciences, applied pharmaceutical sciences, and industrial and clinical pharmacy. The study received approval from the Institutional Review Board (IRB) of Ewha Womans University, with the IRB registration number ewha-202112-0022-03. Participants provided their consent by granting approval to participate in the study. A literature search was performed to gather information on the development status and trends of CBT item types in various license examinations through databases such as PubMed, Google Scholar, RISS, Web of Science, and KoreaMed. Forum presentations at international board committees, media articles, and research reports from the KHPLEI were also included [8–12]. Pre-existing CBT item types used in examinations for healthcare professions were also collected. Multimedia item types were not the focus as they have already been used in some national examinations in South Korea. This study was reviewed by an expert from KHPLEI to improve the appropriateness of data collection and research direction.

### Development process

The following procedure was used to develop Inno-CBT item types:

1. Preparation stage - The research team was provided with a presentation by KHPLEI on previous studies related to typical PBT and CBT item types in national examinations for healthcare professionals and a demand for the development of novel CBT item types. Our research team also collected pre-existing CBT item types from the literature search and organized the websites of various organizations.
2. Development stage - The assignment was given to each researcher to create a draft of novel Inno-CBT item types based on the referenced CBT item types to better suit our needs before attending a workshop session. The research team was divided into four groups, each with a team leader. At the first workshop, the researchers gathered and brainstormed ideas for new features to include in novel Inno-CBT item types. They were encouraged to share their ideas among the groups.
3. Evaluation and summarization stage - During the workshops, the research team discussed the usability and functionality of the draft Inno-CBT item types created by the individual researchers, as well as their applicability to national examinations. Usability refers to the ability of item developers to develop items and users to solve items without technical difficulties. Functionality refers to the extent to which the test items can effectively and correctly measure abilities requested in the exams. At the end of the workshops, each group summarized the newly developed Inno-CBT item types, highlighting key features, including the category of item types, their characteristics, the usability for examinees, advantages and limitations in fidelity, and applicability to KHPLE.

### Feedback and revisions

**Fig. 1:**
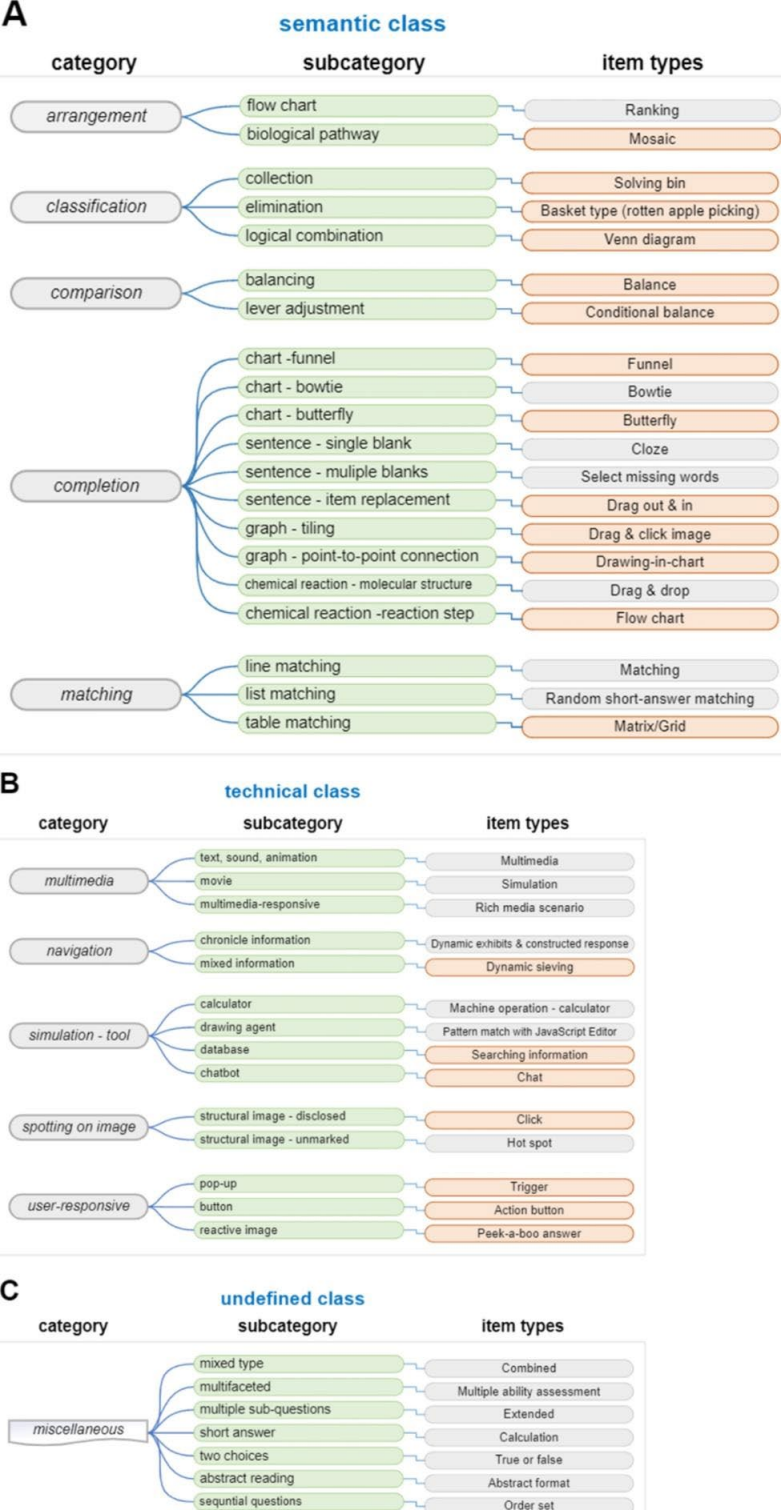
Category of Inno-CBT item types CBT item types were classified into three main classes according to their characteristics: the semantic (**A**), the technical (**B**), and the miscellaneous (**C**). Each class is again composed of a hierarchical structure of categories and subcategories, and item types corresponding to each subcategory are presented. (**A**) The semantic class is composed of ‘arrangement’, ‘classification’, ‘comparison’, ‘completion’, and ‘matching’ categories. (**B**) The technical class includes ‘mutimedia’, ‘navigation’, ‘simulation – tool’, ‘spotting on image’, and ‘user-responsive’ categories. (**C**) The miscellaneous class item types that did not

Refinement was achieved iteratively through feedback and revision processes. Feedback was provided by individual groups or the entire team during workshops. The applicability of the item types to the KHPLE was further evaluated by reviewing reports from KHPLEI [8–12], and the findings were applied to optimize the item types. Finally, the item types were named and categorized based on the innovative target concepts of each type (Fig. 1).

## Results

### Development of novel Inno-CBT item types

We collected 21 pre-existing CBT item types and 20 newly developed Inno-CBT item types. To ensure comprehensive understanding, we classified these 41 CBT item types based on their characteristics (See Supplement Table 1, which thoroughly describes the 41 item types identified and developed, along with examples). To classify the item types, we identified commonalities from various characteristics and organized them into a hierarchical structure.

At the highest level, we divided the item types into three classes: the ‘semantic’ class, the ‘technical’ class, and the ‘miscellaneous’ class. The ‘semantic’ class consists of item types that reflect the types of ability being measured, while the ‘technical’ class is based on the skills characteristically used in CBT item types. Item types that did not belong to these two categories were classified as ‘miscellaneous’.

Each class was further divided into a hierarchical structure of ‘category’ and ‘subcategory’, and the corresponding ‘item types’ were presented in Fig. 1. The ‘semantic’ class includes categories such as ‘arrangement’, ‘classification’, ‘comparison’, ‘completion’, and ‘matching’, which are grouped according to the type of abilities being measured. These categories were further divided into subcategories that describe the implementation method of the items. The ‘technical’ class includes categories such as ‘multimedia’, ‘navigation’, ‘simulation – tool’, ‘spotting on image’, and ‘user-responsive’, which are grouped according to the types of technology used. These categories were also further subdivided into subcategories indicating the details of the technology. For example, the ‘matching’ category in the ‘semantic’ class has three subcategories: line matching, list matching, and table matching. The three CBT item types related to these subcategories were named ‘matching’, ‘random short-answer matching’ and ‘matrix/grid’, respectively.

We found that the pre-existing CBT item types include simple question types such as ‘bowtie’, ‘calculation’, ‘true or false’ and ‘matching’, as well as more complex question types such as ‘rich media scenario’. Generally, most of the pre-existing item types have been used as assisted-learning supporting tools through a web-based program within a learning management system (LMS). Interestingly, most of the challenging item types have rarely been used in national examinations. The most widely implemented item type among them was the ‘multimedia’ item type.

### Characteristics of the inno-CBT item types

The newly developed Inno-CBT item types offered various features reflecting the transformation in the healthcare delivery system and a broad range of innovative environmental changes in healthcare. (Detailed descriptions and examples of all item types can be found in the Supplementary Data). The ‘dynamic sieving’ and ‘searching information’ item types have advantages in measuring the ability to deal with healthcare information data. The ‘dynamic sieving’, especially, assesses the skills to identify and recognize the pertinency of information to resolve problems. The ‘searching information’ item type, which is similar to ‘dynamic sieving’ but requires an internet web surfing environment, can measure internet browsing skills to find information selectively by extracting unrequired information from target information sites.

Some item types were developed to reflect digital healthcare environments. For example, the ‘chat’ item type simulated a digital technique-driven conversation, like a chatbot. This item type presumably could happen between patients and healthcare professionals. The ‘rich media’ was another novel item type that requires an interactive response technique. It was developed to evaluate the ability to cope with risks in patient care procedures.

Other item types were developed to assess the level of understanding of experimental principles, interpretation of laboratory results, and treatment procedures. These examples were ‘action button’, ‘conditional balance’, ‘dynamic exhibits and constructed response’, and ‘solving bin’.

In addition, certain item types were designed to measure the conceptual knowledge of healthcare sciences using simple shapes. One such item type, ‘balance’, was specifically designed to measure the ability to comprehend the concept of benefit and risk, prioritize decision-making processes, evaluate potency equivalency, and assess cost-effectiveness. The ‘mosaic’ item type was developed to assess the ability to find a key puzzle piece to complete the whole picture, such as identifying a chemical compound to complete a biochemical reaction cycle of a human physiological process. Moreover, this item type could be applied to assess the ability of examinees to find a required medication or treatment as a step-by-step process following treatment algorithms. The item types of ‘bowtie’, ‘funnel’, and ‘venn diagram’ were developed to evaluate the problem-solving abilities in determining related components of before and after, cause and outcome, and hierarchical structures by recognizing conceptual diagrams. These item types can help assess the ability to analyze complex situations and understand the relationships between different elements in a system.

In addition, a variety of option styles were introduced in Inno-CBT item types, such as ‘drop-down (cloze)’, ‘drag and drop’, ‘drag and click’, and ‘drag out and drag in’. These different option styles can add diversity and interactivity to the exam, as well as assess different cognitive skills such as attention to detail and spatial reasoning.

## Discussion

The goals of national license examinations are to test the core knowledge, skills, and behaviors of professionals new to their practice in South Korea. Those practices were included in different ways, like readiness for safe practice, managing uncertainty, and delivering person-centered care. Thus, the examinations should be up to date to make sure they reflect real-life, day-to-day medical practices. In that sense, novel item types are needed, and examinations should be applied with any perspective of test developers’ intention to measure the capability of the test takers. Such item types are useful when assessing complex competencies, such as clinical practice, MCQs are limited in the amount of information obtained about an examinee’s understanding of some phenomena. In addition, MCQs are not realistic portrayals of activities that occur in practice, so novel item types may better assess the myriad of actual activities that occur in practice.

We developed 41 Inno-CBT item types and incorporated novel and innovative tools that can assess the skillsets and knowledge readiness of entry-level healthcare professionals. Key development procedures included identifying needs for new item types, creating and adopting innovative features, designing prototypes, refining and revising, and defining item types [13, 14].

Healthcare has been going through major digital transformations due to the extensive amount of information and social communication media technologies [15]. Using mobile healthcare apps, electronic health records (EHRs), and professional organization websites has become a daily routine practice. Under the circumstances, action to utilize and sort out required information for problem solving is frequently tentative in nature; however, healthcare professionals should cultivate the ability to discern and select core information confidently.

Our main goal was to develop item types reflecting recent changes in healthcare practices, where delivery methods such as multiple-choice questions (MCQs) were unsuitable, and ultimately implement new item types to the KHPLE. Thus, the item types of ‘dynamic sieving’ and ‘searching information’ were prioritized by our research team to be employed in the KHPLE. Moreover, the item type of ‘chat’ was also recommended due to its high applicability in assessing virtual communication ability in the era of digital transformation of the healthcare environment. Sharing opinions and making conversation using texting tools have been widely used since they are quick and simple. Furthermore, the effectiveness has been emphasized, especially due to social distancing from the recent pandemic [16] and the public demand for remote care for isolated individuals living alone [17]. This was consistent with the study by de Oliveira et al. (2021), in which text messages were useful tools to support people with suspected COVID-19 who experienced home isolation [17].

Previously, the ‘multimedia’ item type had been applied in the KHPLE [12] and other countries [18, 19]. The fidelity of the examination was improving by incorporating images, sounds, animations, and video clips. The item types with high fidelity were also considered with an item type of ‘hot spot’ over a simple ‘click’ because of the presence of ‘distractors’ in a ‘hot spot’ item. For example, examinees are tasked with selecting the exact location of an arthritic lesion on a knee x-ray screen, while a computer screen senses examinees’ cursor locations at every part of the image.

There were some item types that could be considered for the national examinations. A ‘machine-operated’ item type required an operational skill-reproducing technology. Despite the technological support needed, this item type directly assessed hands-on skills (e.g., manipulating a calculator, an electrocardiogram monitoring device, and a defibrillator).

In addition, the item types of ‘flow chart’, ‘grid/matrix’, and ‘ranking/ordering’ were also identified for potential in the national examinations to measure knowledge of sequencing orders or prioritizing medical procedures. The ‘bowtie’ and ‘matrix’ could be used similarly to assess problem-solving. However, these item types may require scoring protocols for partial or weighted points, and controlling difficulty level depends on the number of multiple correct answers. Item types of ‘abstract formats’ and ‘order set’ had advantages for measuring comprehensiveness in clinical reasoning. For example, ‘order set’ evaluated the ability to solve procedural sequences, which is a crucial skill for health professionals, but these types of items had low priority to be implemented due to time constriction allocated for a question in the national examination.

Type A option items (MCQs) have been the most common item type in the national examinations in South Korea. In this study, we proposed different option styles, like ‘drag and drop’, ‘drag in and drag out’, ‘drop-down (cloze)’, and ‘marking’, as well as a simple ‘click’ option. These styles would prevent from scoring a lucky guess.

Furthermore, add-on features of ‘combination’ and ‘extended’ item types provided freedom in expansion and increased applicability of existing item types. A ‘combination’ item type was constructed with the combination of two or more item types, and an ‘extended’ item type was the item type allowing a case scenario in the stem (question part of an item). For example, the ‘extended rich media’ item type consisted of a ‘rich media’ containing a case script, and a ‘combination rich media sound’ included the features of a ‘rich media’ and a sound file of a ‘multimedia’ item type. Moreover, because the various types of the stem could be mixed and matched with different styles of options (a listing of answers to be chosen) in many ways, Inno-CBT item types could provide additional item types with high flexibility to be fitted to the purpose of test questions.

### Barriers

#### Difficulty level and testing time

The shift to Inno-CBT item types from PBT and traditional CBT item types could increase the difficulty for users due to content complexity and unfamiliar user technology of computer operation [20]. The content complexity could result from the item types, including lengthy cases, a series of sub-questions, more than one correct answer, and a calculation tool installation to solve questions. In addition, examinees might complain of being fatigued with spending extra time to solve test questions because they may face different kinds of item types. It is necessary to control item difficulty, discrimination, and discernment when applying Inno-CBT to a test question.

#### Scoring system

Scoring protocols of currently available CBT items use a conventional number right scoring method. Correct answers are scored with a positive value, incorrect answers and absent or omitted answers with a value of zero based on the type A MCQs in South Korea. A major concern of the scoring protocol was that students could answer correctly through guessing. Inno-CBT item types may reduce the chance of guessing the correct answer; however, the concerns about adopting a fair scoring system with a variety of item types still remain. Importantly, scoring rubrics and protocols of Inno-CBT item types should be developed considering different types, difficulty levels, and the number of correct answers for each item type. Automatic score marking protocols also need to be prepared for immediate test results after scoring as all or none, partial, or different weighted scoring, which was mentioned with similar perspectives by the National Council Licensure Examination for Registered Nurses in the US [21].

#### Implementation technology

We found that the infrastructures of technical support to implement new item types were very lacking. For example, ‘chat’ requires the technology of showing an input text followed by an output response in alternating manners; ‘hotspot’ requires a recognition technique of a cursor pointing to a location on the computer screen; ‘searching information’ requires accessibility and retrieval credentials to data servers while maintaining a high level of information security to avoid any fraudulent events; and ‘rich media’ and ‘simulation’ require IT support to operate a broad range of digital interactive media within a scenario. Several commercial solutions were found, such as an LMS for primary and secondary education [22, 23], but not for healthcare professional examinations.

#### Future suggestions

For a smooth implementation of Inno-CBT item types, the reliability and suitability of each item type need to be validated, and quality assurance should be continued. The prioritization of a gradual implementation of item types to national examinations should be considered. Moreover, education sessions for the item writers need to be provided to introduce structures, characterizations, and the targeted ability to assess individual item types. In addition, a computer platform enabling the item writers to experience the building demo items would be essential. Moreover, a handbook for how to prepare Inno-CBT tests and tutorial websites to practice maneuvering techniques are needed to be provided to test takers.

The investment in building up a sustainable item development system and system management companies is an imminent issue. Further, securing the confidentiality of items and internet safety during examinations should be guaranteed. In addition, server capacity to manage the heavy connection traffic during a test should be reserved. A pilot study is warranted to apply the Inno-CBT item types in the near future. Such a study would evaluate the feasibility of the Inno-CBT item types by managing the difficulty of the test, scoring system, and test security.

Some limitations are present in the study. Qualitative analysis was not employed during the evaluation and summarization stage, but the research team dedicated considerable effort to engage in comprehensive discussions and reviews to evaluate the newly developed item types. Another limitation would be the study only included the faculty members of pharmacy schools. Nonetheless, the development process involved a collaborative effort among internal and external experts specializing in the field of from various healthcare professionals, including the faculty of medicine.

## Conclusions

We developed novel Inno-CBT item types for assessments in national licensure examinations. These Inno-CBT item types can contribute to measuring the competencies and capabilities of entry-level healthcare professionals in a multifaceted and in-depth manner. It should be noted that sufficient validity and reliability should be established prior to implementing the item types in the national examinations.

### Electronic supplementary material

Below is the link to the electronic supplementary material.

Supplementary Material 1

## Data Availability

All data generated or analyzed during this study are included in this published article.

## Abbreviations

CBT: Computer-based test
EMR: Electronic medical record
HER: Electronic health record
ICT: Information and communication technology
Inno-CBT: Innovative computer-based test
IT: Information technology
KHPLE: Korea Health Personnel Licensing Examination
KHPLEI: Korea Health Personnel Licensing Examination Institute
LMS: Learning management supportive system
MCIT: Multiple choices item type
PBT: Paper-based test
