# Supplementary material for: Novel innovative computer-based test (Inno-CBT) item types for national licensing examinations for health care professionals
Source: BMC Med Educ. 2023 Aug 9;23:560. doi: 10.1186/s12909-023-04444-5 (PMC10410778; doi:10.1186/s12909-023-04444-5)
Supplement: Supplementary file 1 — Supplementary Material 1 [file 12909_2023_4444_MOESM1_ESM.docx]

Supplementary data

**Novel innovative computer-based test (Inno-CBT) item types for national licensing examinations for health care professionals**

Kwang-Hoon Chun, PhD^1†^, Hye Kyung Jin, PhD ^2†^, Jeong-Hyun Yoon, PharmD ^3^, Myeong Gyu Kim, PhD ^4^, Kyung Hee Choi, PhD ^5^, Eunyoung Kim, PharmD, PhD ^6^, Hyunah Kim, PharmD ^7^, Jin-Ki Kim, PhD ^8^, Gyudong Kim, PhD ^9^, Kyungim Kim, PhD ^10^, Ju-Yeun Lee, PhD ^11^, Eun Kyoung Chung, PharmD, PhD ^12^, Young Sook Lee, PharmD ^13*^, Sandy Jeong Rhie, PharmD, PhD ^14*^

**Co-1^st^ authors:**

1 Kwang-Hoon Chun, PhD

Affiliation: Gachon Institute of Pharmaceutical Sciences, College of Pharmacy, Gachon University, Incheon, Korea

Email: khchun@gachon.ac.kr

2 Hye Kyung Jin, PhD

Affiliation: College of Pharmacy, Dongguk University-Seoul, Goyang, Korea

Email: hkjin@dongguk.edu

**Co-authors:**

3 Jeong-Hyun Yoon, PharmD

Affiliation: College of Pharmacy, Pusan National University, Busan, Korea

Email: jyoon@pusan.ac.kr

4 Myeong Gyu Kim, PhD

Affiliation: College of Pharmacy and Graduate School of Pharmaceutical Sciences, Ewha Womans University, Seoul, Korea.

Email: kimmg@ewha.ac.kr

5 Kyung Hee Choi, PhD

Affiliation: College of Pharmacy, Gachon University, Incheon, Korea

Email: [khchoi@gachon.ac.kr](mailto:khchoi@gachon.ac.kr)

6 Eunyoung Kim, PharmD, PhD

Affiliation: Department of Health, Social and Clinical Pharmacy, College of Pharmacy, Chung-Ang University, Seoul, Korea

Email: eykimjcb777@cau.ac.kr

7 Hyunah Kim, PharmD

Affiliation: College of Pharmacy, Sookmyung Women’s University, Seoul, Korea

Email: [hyunah@sookmyung.ac.kr](mailto:hyunah@sookmyung.ac.kr)

8 Jin-Ki Kim, PhD

Affiliation: College of Pharmacy, Institute of Pharmaceutical Science and Technology, Hanyang University, Ansan, Korea

Email: jinkikim@hanyang.ac.kr

9 Gyudong Kim, PhD

Affiliation: College of Pharmacy, Chonnam National University, Gwangju, Korea

Email: gdkim0217@jnu.ac.kr

10 Kyungim Kim, PhD

Affiliation: College of Pharmacy, Korea University, Sejong, Korea

Email: kim_ki@korea.ac.kr

11 Ju-Yeun Lee, PhD

Affiliation: College of Pharmacy and Research Institute of Pharmaceutical Sciences, Seoul National University, Seoul, Korea

Email: jypharm@snu.ac.kr

12 Eun Kyoung Chung, PharmD, PhD

Affiliation: Department of Pharmacy, College of Pharmacy, Kyung Hee University, Seoul, Korea.

Email: [cekchung@khu.ac.kr](mailto:cekchung@khu.ac.kr)

**Corresponding authors**

13 Young Sook Lee, PharmD

Affiliation: College of Pharmacy, Keimyung University, Daegu, Korea

Email: [youngslee@kmu.ac.kr](mailto:youngslee@kmu.ac.kr)

Tel: +82.53.580.6657

14 Sandy Jeong Rhie, PharmD, PhD

Affiliation: College of Pharmacy and Graduate School of Pharmaceutical Sciences, Ewha Womans University, Seoul, Korea

Email: [sandy.rhie@ewha.ac.kr](mailto:sandy.rhie@ewha.ac.kr)

Tel: +82.2.3277.3023

^†^First author: Kwang-Hoon Chun, Hye Kyung Jin

These two authors equally contributed to this work.

## ^*^Corresponding author: Young Sook Lee, Sandy Jeong Rhie

**Supplement Table 1. Characteristics of the Newly Developed Inno-CBT Item Types**

| **No** | **Category** | **Name** | **Description** | **Example** |
| --- | --- | --- | --- | --- |
|  | **Subcategory** |  |  |  |
| 1 | arrangement | Mosaic | **Characteristics:** To find a piece to solve a test question to complete a final product. For example, it may apply to a metabolic pathway, biochemical cycle, or mechanism of action process by selecting a correct element and filling in the blank.  **Advantages:** It is easy to answer intuitively with a mosaic presentation. The level of difficulty can be controlled using the number of options to answer the question.  **Limitations:** Controlling difficulty levels and a partial scoring system.  **Reference:** Developed by the study team. | 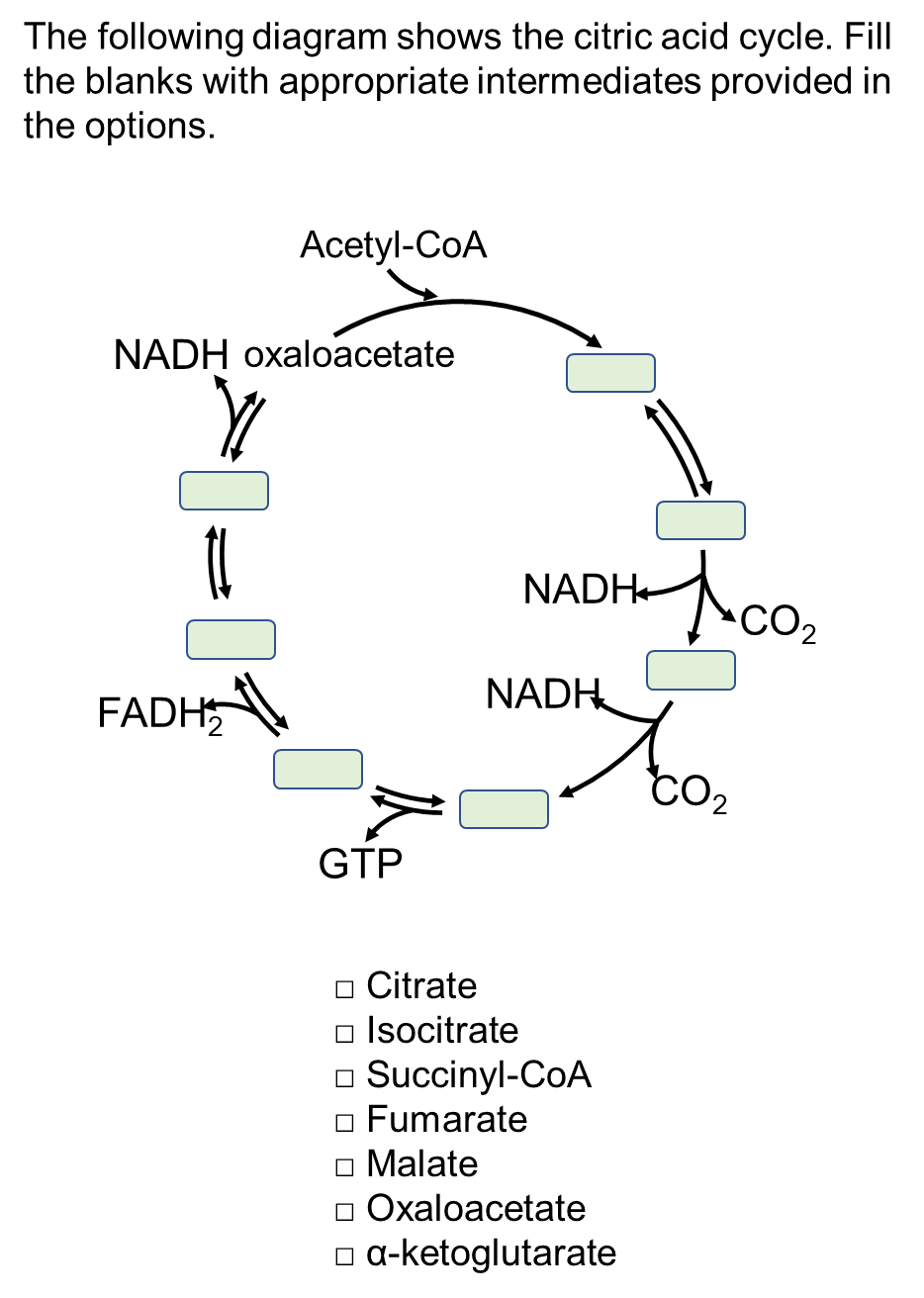 |
|  | biological pathway |  |  |  |
| 2 | arrangement | Ranking | **Characteristics:** To arrange randomly placed content in a test question in order by dragging options. It evaluates the ability to prioritize various content or actions encountered by healthcare professionals. It can measure if examinees are aware of the correct order, which is hard to test in multiple choice item type.  **Advantages:** It is easy to answer intuitively with a charting presentation. The level of difficulty can be controlled using the number of options to answer the question.  **Limitations:** A scoring system needs to be developed to allocate partial points.  **Reference:** Drag & drop ranking question type. https://help.alchemer.com/help/drag-drop-ranking. Accessed on January 15, 2022. | 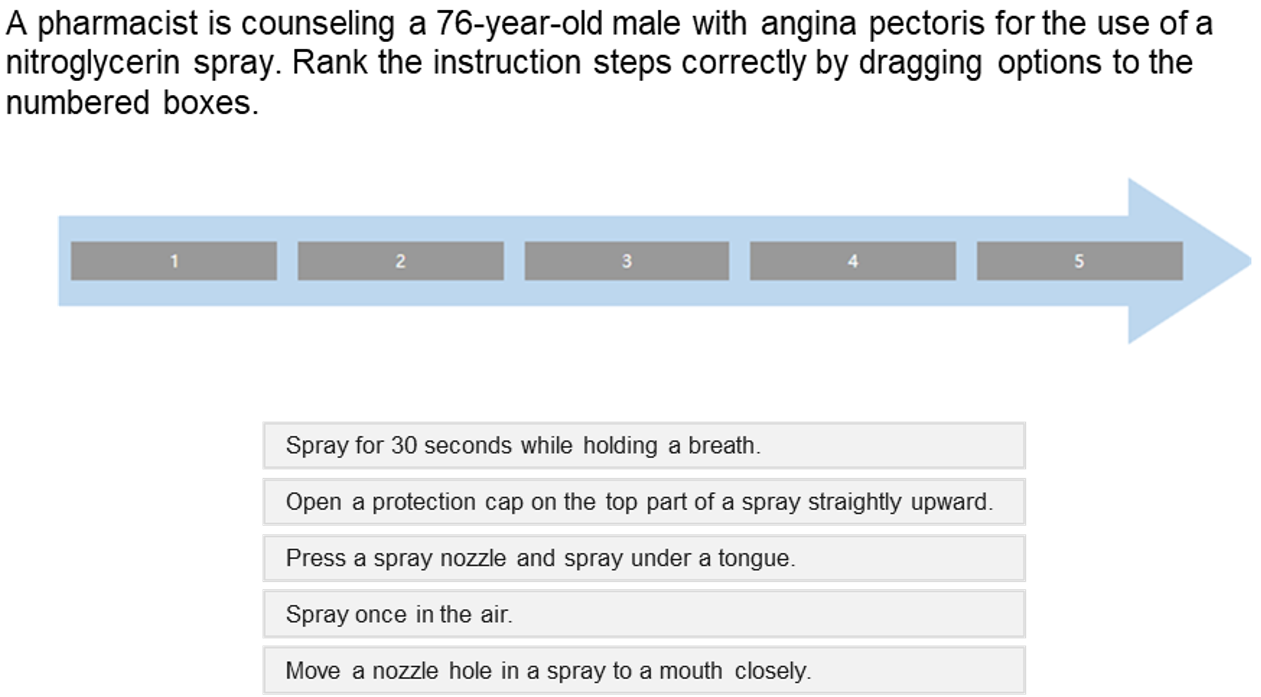 |
|  | flow chart |  |  |  |
| 3 | classification | Basket type, rotten apple picking | **Characteristics:** To measure the ability of selecting the unmatched content or misinformation among the mixed content in the basket.  **Advantages:** It can evaluate the ability to select unnecessary information to perform a service.  **Limitations:** Need to carefully form a clear answer that does not become an argument if the examinee selected the inappropriate answer.  **Reference:** Developed by the study team. | 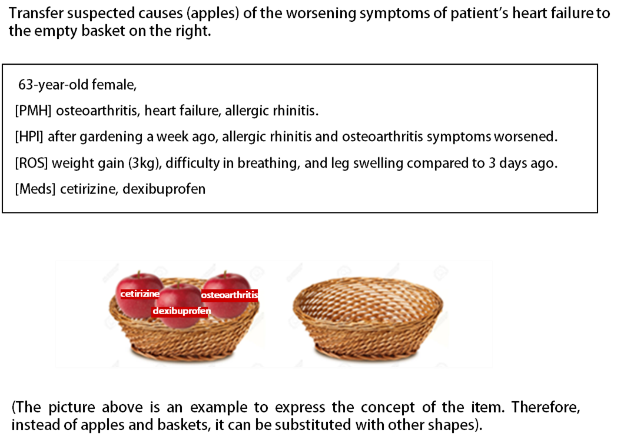 |
|  | elimination |  |  |  |
| 4 | classification | Solving bin | **Characteristics:** Filling a hole in a box with correct responses that meet the presented conditions. It consists of two steps: 1) selecting one item with the characteristics described in the question and 2) placing the selected item in an option expressed as a bin by dragging.  **Advantages:** It can focus on the assessment of the examinees’ level of understanding of experimental principles, interpretation of laboratory results, and treatment procedures.  **Limitations:** Requires the technical support to drop the answers into the hole of the respective shape.  **Reference:** Developed by the study team. | 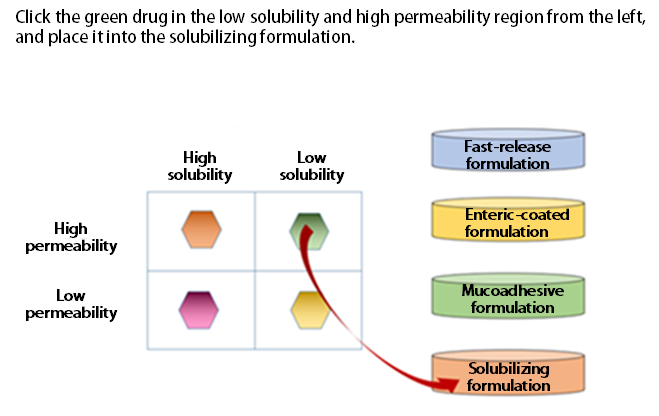 |
|  | collection |  |  |  |
| 5 | classification | Venn diagram | **Characteristics:** It assesses the ability of understanding the hierarchical interrelationship of various concepts under one theme. It requires positioning each item in a chosen shape in the Venn diagram, such as a drug-related problem in a big band, and drug adverse events and drug use errors in small bands.  **Advantages:** It visualizes item types for easy intuitive responses.  **Limitations:** It may limit topics to be developed. The scoring system needs to be discussed.  **Reference:** Developed by the study team | 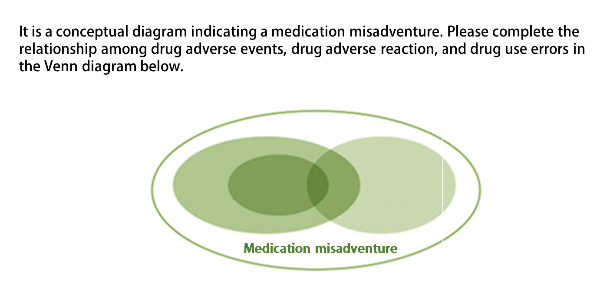 |
|  | logical combination |  |  |  |
| 6 | comparison | Balance | **Characteristics:** To measure the ability of understanding the concept of benefits and risks or priority of a decision-making process, potency equivalency, and cost-effectiveness. One item is placed on one side of the scale, then examinees select another comparable item to put on the other side of the scale.  **Advantages:** It is easy to understand as it is presented schematized. It evaluates comparative values rather than the concept of right or wrong in healthcare practice.  **Limitations:** It may be similar with type A MCQs in selecting an answer.  **Reference:** Developed by the study team. | 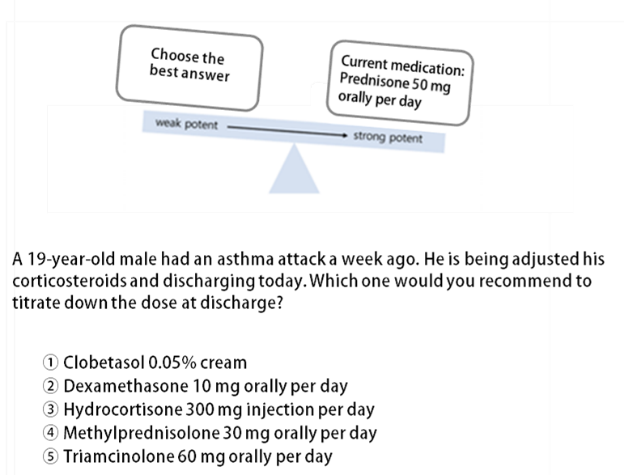 |
|  | balancing |  |  |  |
| 7 | Comparison | Conditional balance | **Characteristics:** It requires that examinees infer the prerequisite conditions from given results in a certain experimental condition. It applies the selected condition in balance to measure the ability to understand cause-effect analysis and experimental principles.  **Advantages:** It visualizes the relationship between experiment results and conditions, and it evaluates the process of interpreting results and logical reasoning.  **Limitations:** Requires the implementing technique to operate the questions.  **Reference:** Developed by the study team. | 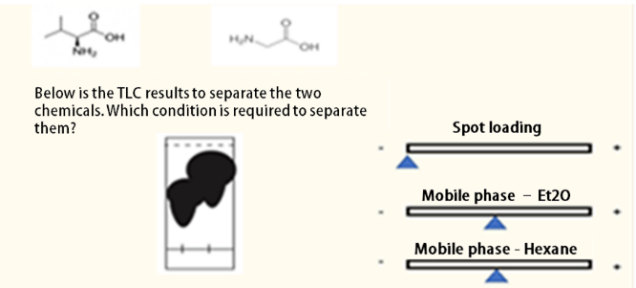 |
|  | lever adjustment |  |  |  |
| 8 | completion | Bowtie | **Characteristics:** It has a bowtie-shaped template, which consists of five parts (four bows and one center knot). A major event (i.e., disease, diagnosis, etc.) is located in the center knot part of a bowtie-shaped diagram, and a cause or background element of the event is placed on the left bow. Outcomes or resolution of the event are placed on the right bows. The examinees are asked to fill in blank(s) from one of five parts of the bowtie. It measures the ability to determine and structure causes, therapies, and treatments based on comprehensive understanding throughout the clinical process.  **Advantages:** It can be used for case type and problem-solving questions. It can evaluate comprehensive and integrated clinical knowledge rather than fragmented memorization-type knowledge.  **Limitations:** It would take a long time to solve. The difficulty level can increase depending on the number of answers required to solve the questions.  **Reference:** Phil D. Next Generation NCLEX Update. https://www.ncsbn.org/public-files/presentations/2021_MYM_pdickison_NGN.pdf. Accessed on September 15, 2021. | 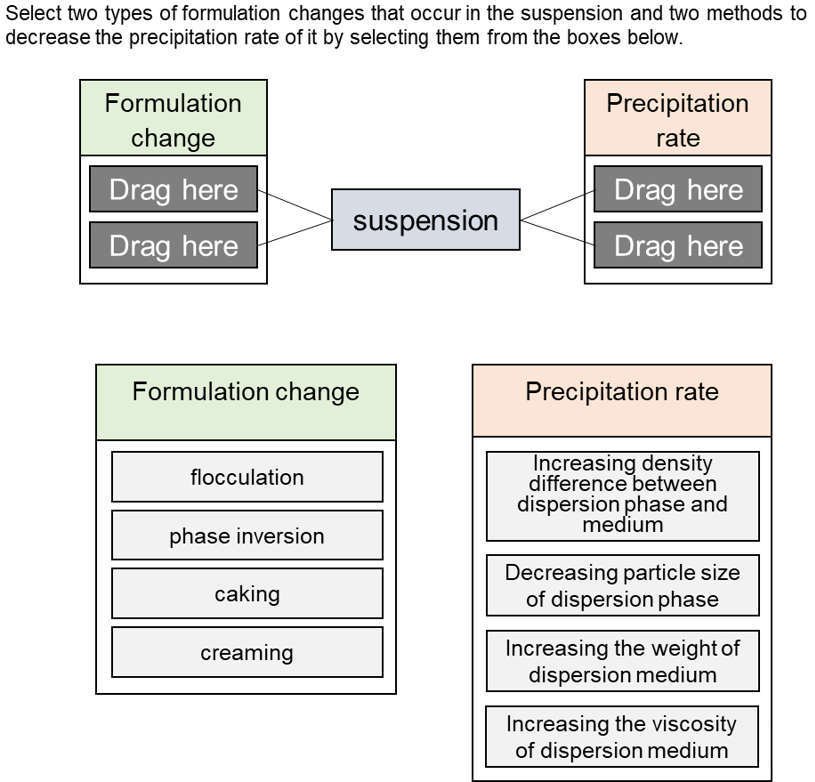 |
|  | chart-bowtie |  |  |  |
| 9 | completion | Butterfly | **Characteristics:** It is similar to the bowtie item type, but it would provide more options to choose. As an example, it displays patient symptoms, signs, and laboratory results on the left wings of the butterfly and places surgery, medication, and follow-up on the right wings. Diagnosis may be placed in the knot part. It asks examinees to fill in the blanks with the correct answer.  **Advantages:** It is possible to simplify a case type lengthy question using each concept circle.  **Limitations:** The level of difficulty needs to be controlled when the question asks more than one concept circle.  **Reference:** Developed by the study team. | 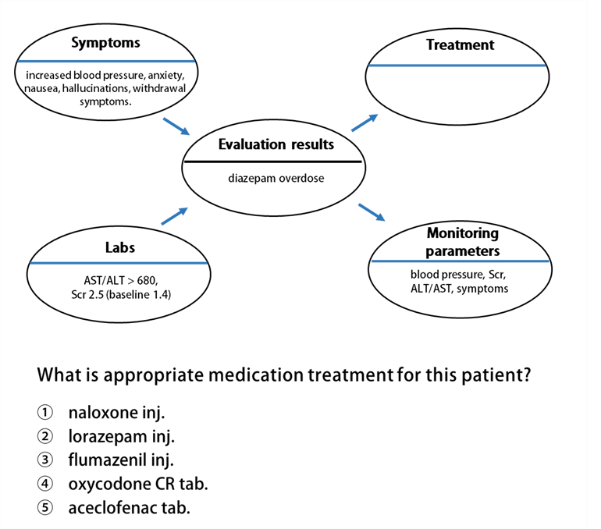 |
|  | chart-butterfly |  |  |  |
| 10 | Completion | Cloze | **Characteristics:** It provides options in a drop-down format in the form of words, sentences, and images. It measures the ability to choose the single best or multiple answers among options.  **Advantages:** It can reduce spatial distraction because there are no options separately listed on the screen.  **Limitations:** The level of difficulty can be increased if there is more than one drop-down in one question.  **Reference:** Moodle. Embedded Answers (Cloze) question type. https://docs.moodle.org/27/en/Embedded_Answers_(Cloze)_question_type. Accessed on September 15, 2021. | 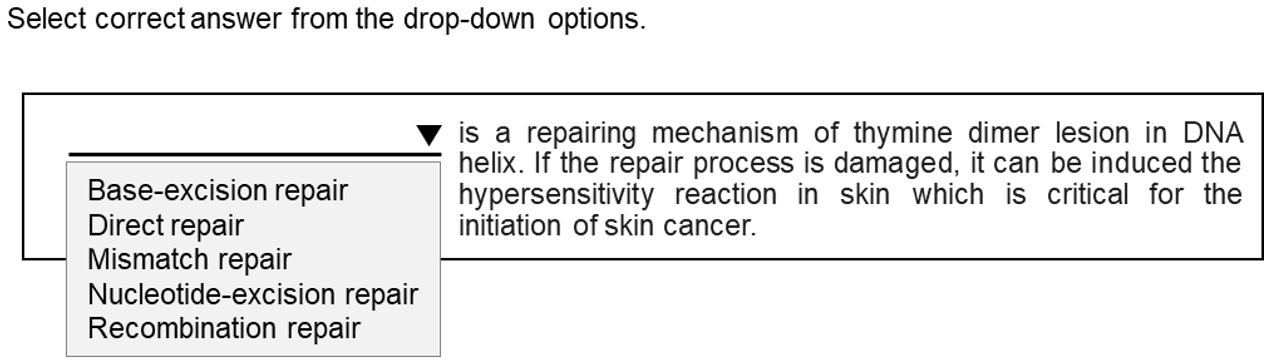 |
|  | sentence-single blank |  |  |  |
|  | lever adjustment |  |  |  |
| 11 | Completion | Drag & click image | **Characteristics:** It requires an additional click action after dragging the response when assessing the ability to point to an issue of interest and connect solutions.  **Advantages:** The questions that are difficult to present due to limitations in expression with descriptive ways can be implemented by image.  **Limitations:** It needs technical support and may take time to solve a question.  **Reference:** Developed by the study team. | 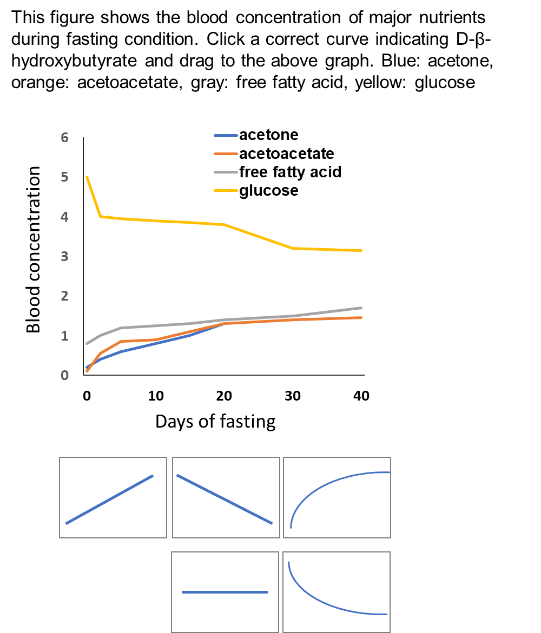 |
|  | graph-tilting |  |  |  |
| 12 | Completion | Drag & drop | **Characteristics:** It requires selecting responses (such as a text, image, or formula) and dragging and dropping them in the designated location.  **Advantages:** It is intuitive and easy to understand a question and locate an answer.  **Limitations:** When a question has more than one answer, the difficulty level should be adjusted, and the allocation of a partial scoring system should be considered.  **Reference:** Moodle: ddclassify Question Type. https://docs.moodle.org/dev/ddclassify_Question_Type. Accessed on September 15, 2021. | 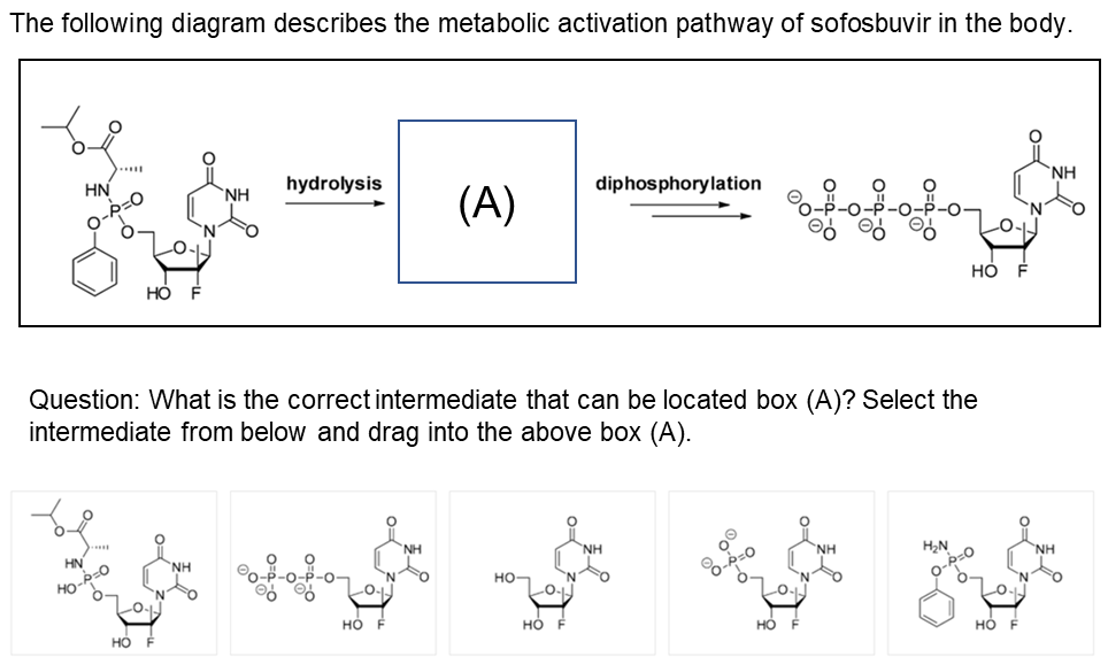 |
|  | chemical reaction-molecular structure |  |  |  |
| 13 | Completion | Drag out & in | **Characteristics:** It requires the action of removing the incorrect information and replacing it with the correct information from given options to solve a test question. It measures both the ability to recognize errors and the ability to suggest the substitutions.  **Advantages:** It can evaluate the ability of problem recognition and solving at the same time.  **Limitations:** It needs technical support to operate, and the scoring system needs to allocate partial points.  **Reference**: Developed by the study team. | 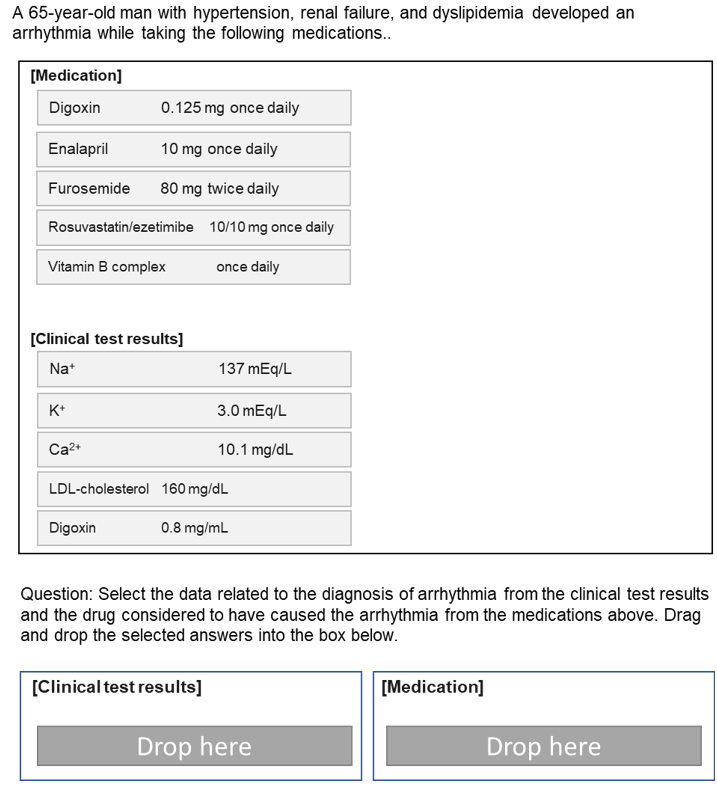 |
|  | sentence-item replacement |  |  |  |
| 14 | Completion | Drawing-in-chart | **Characteristics:** It measures the ability to understand how a value changes over time and generates the graphs. The sample question expresses the changes in numeric values over time by drawing whole or part of a graph.  **Advantages:** It can evaluate the comprehensive understanding of a concept using the graphs compared to the common paper-and-pencil test type.  **Limitations:** It requires technical support to implement the click to initiate the line drawing.  **Reference:** Developed by the study team. | 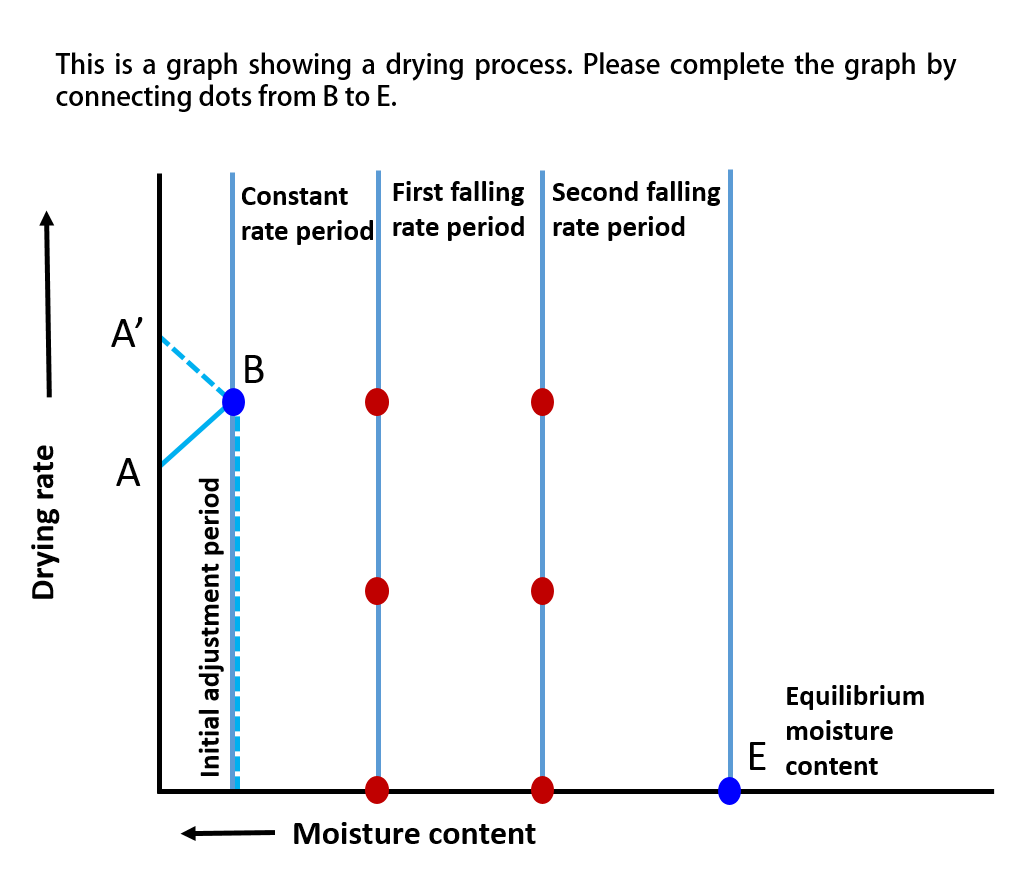 |
|  | graph, point-to-point connection |  |  |  |
| 15 | Completion | Funnel | **Characteristics:** It schematizes the cause and effect of an event in a funnel shape. It asks examinees to select either the correct causes or outcomes and move the response into the funnel by dragging it.  **Advantages:** It is easy to understand using the visualized shape.  **Limitations:** It can be lengthy.  **Reference:** Developed by the study team. | 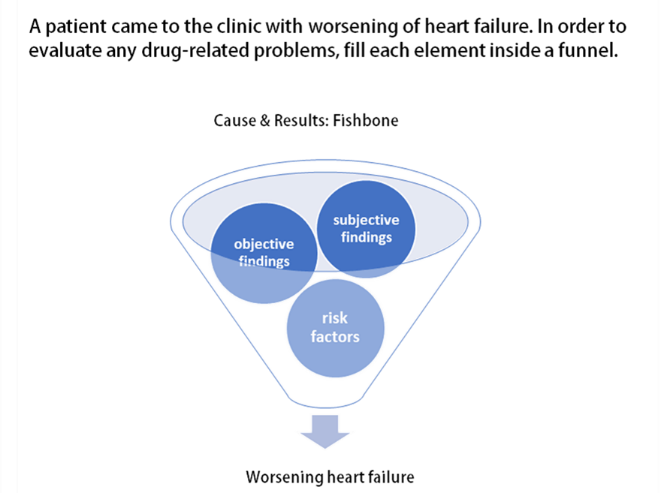 |
|  | chart-funnel |  |  |  |
| 16 | Completion | Flow chart | **Characteristics:** It illustrates tasks or reactions with a step-by-step sequence and asks examinees to choose the answer corresponding to the given step by dragging it in the blank. It measures the understanding of the sequential relationship of a process.  **Advantages:** It evaluates the ability to use knowledge necessary to identify and handle incidents in the healthcare field.  **Limitations:** It requires the independence of each questioned concept since it has at least two answers. A partial scoring system is needed.  **Reference:** Developed by the study team. | 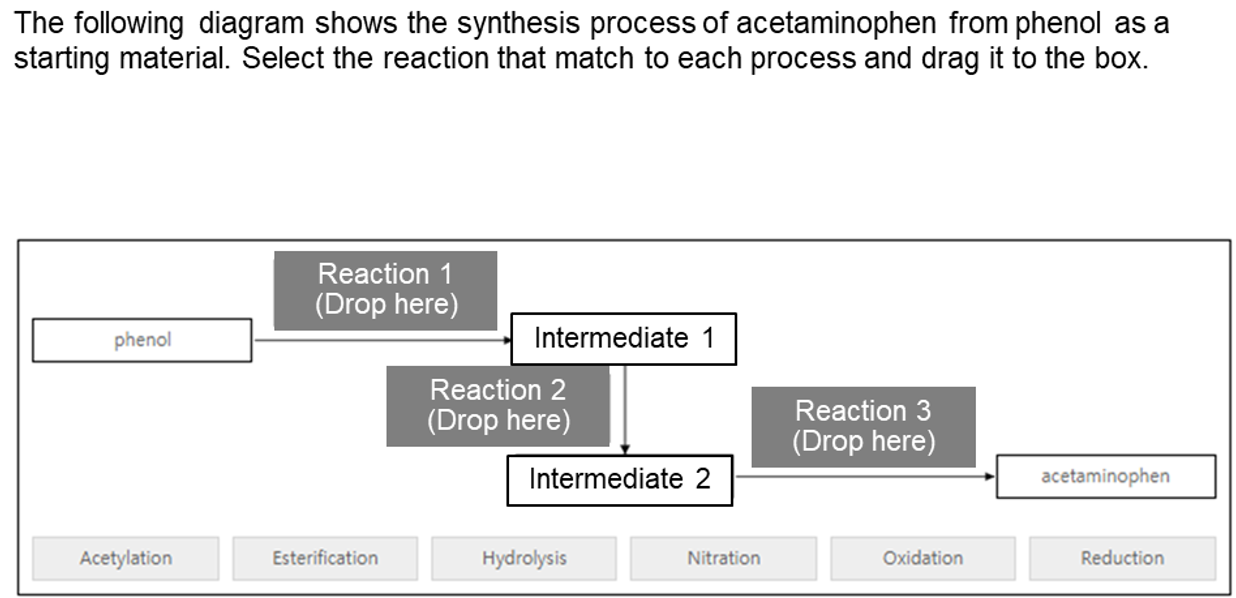 |
|  | chemical reaction-reaction step |  |  |  |
| 17 | Completion | Select missing words | **Characteristics:** It requires reading sentences and finding appropriate words in a blank space using drop-down options to ensure that the sentence’s meaning is correct.  **Advantages:** It is useful when the presented material is lengthy. It saves times to go back and forth between stems and options.  **Limitations:** The time to solve the question needs to be controlled. It may limit the questioning words at one time.  **Reference:** Open Learn Create. Select missing words. https://www.open.edu/openlearncreate/mod/oucontent/view.php?id=52747&section=2.3.6. Accessed on September 15, 2021.  Photo credit: https://docs.moodle.org/401/en/images_en/thumb/1/1c/selectblankdisplay.png/400px-selectblankdisplay.png. | 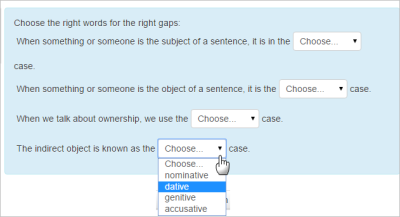 |
|  | sentence-multiple blanks |  |  |  |
| 18 | Matching | Matching | **Characteristics:** It measures the ability of arranging random items properly on either side by connecting them with a line.  **Advantages:** It is easy to understand and is intuitive to solve.  **Limitations:** It may distract the examinee since it may ask several problems at once. The level of difficulty needs to be controlled.  **Reference:** Moodle. Matching question type. <https://docs.moodle.org/401/en/Matching_question_type>. 2021. Accessed on January 15, 2022. | 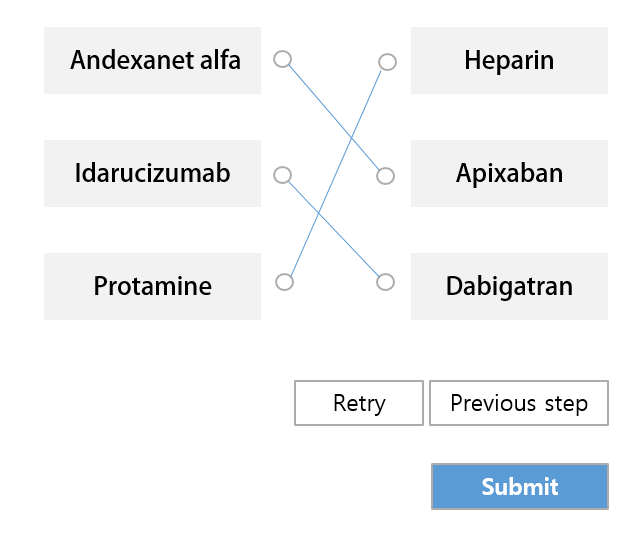 |
|  | line matching |  |  |  |
| 19 | Matching | Matrix/Grid | **Characteristics:** It lists content in rows respectively and places multiple options in columns. It requires marking the correct responses in a meeting point between a row and a column.  **Advantages**: It allows multiple factors on a single topic to be questioned at once.  **Limitations:** Because all boxes need to be marked with the correct answers, a large number of incorrect answers may occur if the accurate concept is lacking.  **Reference:** Saunders comprehensive review for the NCLEX- PN® examination (8th ed.). p.72. https://yourknowledgedigest.files.wordpress.com/2020/04/saunders-comprehensive-review-for-the-nclex-rnc2ae-examination-8th-edition.pdf. Accessed on January 15, 2022. | 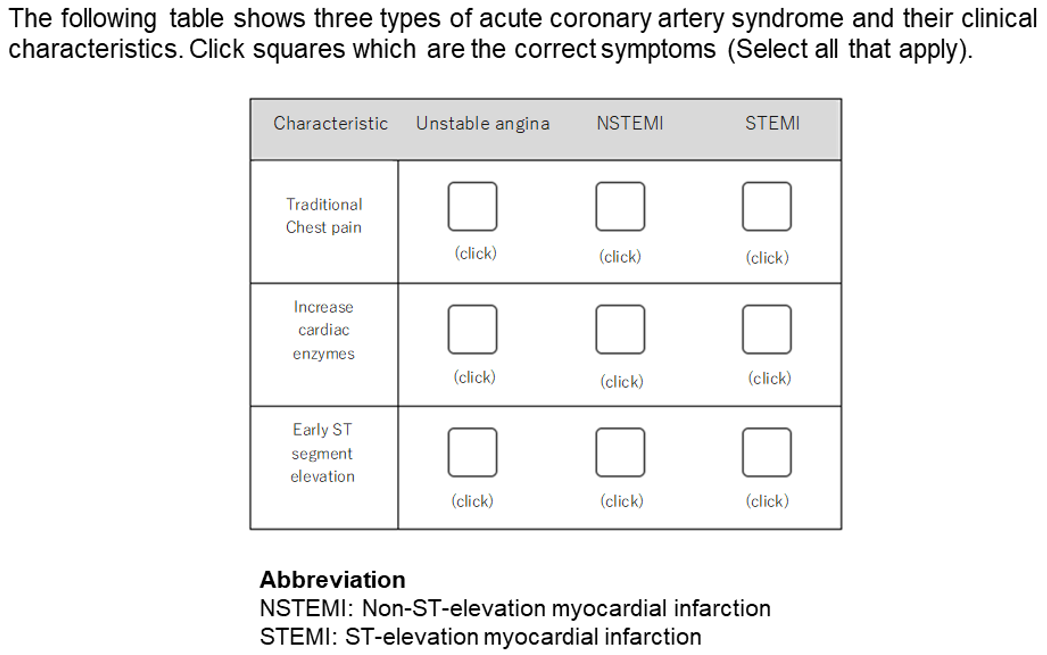 |
|  | table matching |  |  |  |
| 20 | Matching | Random short-answer matching | **Characteristics:** Short-answer questions on each line and asks examinees to choose the correct answer one by one. Examinees must solve all included matching questions with drop-down options.  **Advantages:** It can be easy to find how to operate the item type intuitively using drop-down operation.  **Limitations:** It should be questioned under one theme and options can be shared. The scoring system needs to be developed to decrease points based on the number of trials with incorrect answers.  **Reference:** Moodle. Random Short-Answer Matching question type. https://docs.moodle.org/27/en/Random_Short-Answer_Matching_question_type. Accessed on September 15, 2021. | 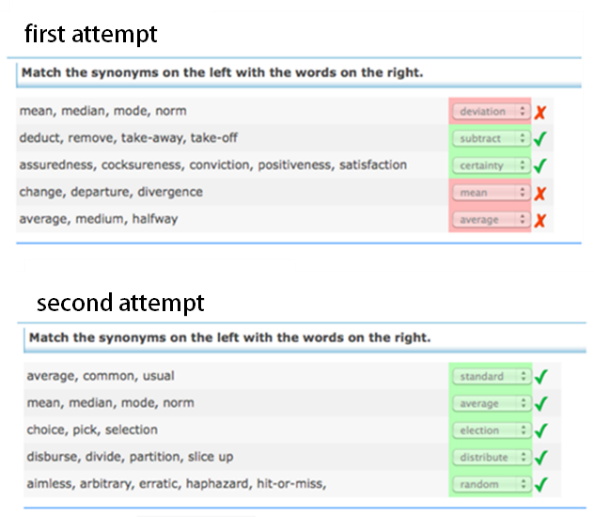 |
|  | list matching |  |  |  |
| 21 | Multimedia | Multimedia | **Characteristics:** It is a widely used form of CBT with audiovisual materials, such as text, sounds, images, graphics, animations, video clips, three-dimensional representation, or infographics.  **Advantages:** It reflects the clinical practice setting using a variety of multimedia tools. It can be applied in combination with other item types.  **Limitations:** It requires the development of multimedia materials to use in the item types. **Reference:** Saunders comprehensive review for the NCLEX- PN® examination (8th ed.). p.64. E-book. <https://yourknowledgedigest.files.wordpress.com/2020/04/saunders-comprehensive-review-for-the-nclex-rnc2ae-examination-8th-edition.pdf>. Accessed on January 15, 2022. Step 2. Accessed on January 15, 2022.Photo credit: Aseptic technique Errors and omissions clips. https://www.youtube.com/watch?v=rSE-nKFzS-Q&list=PLN-7v_nLPMpy7xggAhDEj1C6GgLrDSxHb&pp=iAQB, 2019. Accessed on January 15, 2022. | 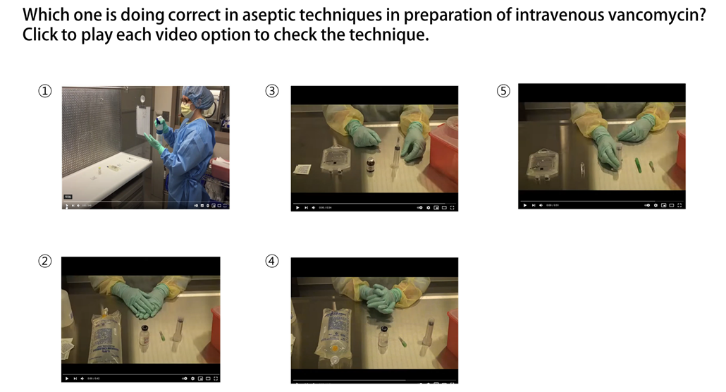 |
|  | text, sound, animation |  |  |  |
| 22 | Multimedia | Simulation | **Characteristics:** It simulates various situations by audio and video clips that health-care personnel may encounter in actual clinical or experimental situations.  **Advantages:** It can measure clinical performance, which is difficult to evaluate using PBTs.  **Limitations:** It requires technical support, multimedia clips and inserts, and a play time limit per insert.  **Reference:** Elsevier Education. Shadow Health(R). https://evolve.elsevier.com/education/simulations/shadow-health. Accessed on January 15, 2022. Photo credit: Eliane Alhadeff. Serious game market. Virtual patient serious games market expects 21% CAGR Over The Period 2014-2019. https://www.Seriousgamemarket.com/2014/07/virtual-patient-serious-games-market.html. Accessed on January 15, 2022. | 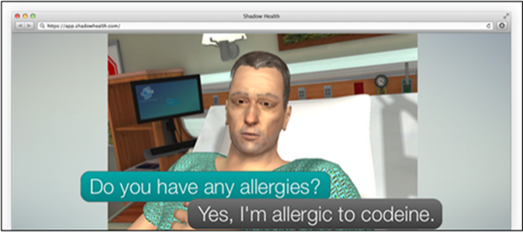 |
|  | video |  |  |  |
| 23 | Multimedia | Rich media scenario | **Characteristics:** It provides an interactive experience and a vignette, such as a clinical case presentation, in a multimedia format without text. It requires pointing and clicking specific parts of the reactive media using a mouse to receive a response. It is similar to the multimedia item type in that it uses video, illustration, sound, etc. However, there is a difference in the items to which examinees respond by moving the mouse.  **Advantages:** It reflects clinical situations closely.  **Limitations:** It needs multimedia clips and materials to develop the item type and technical support.  **Reference:** Saunders comprehensive review for the NCLEX- PN® examination (8th ed.). p.56, 93. E-book. https://yourknowledgedigest.files.wordpress.com/2020/04/saunders-comprehensive-review-for-the-nclex-rnc2ae-examination-8th-edition.pdf. Accessed on January 15, 2022. Step 2. Accessed on January 15, 2022.  Photo credit: https://slideplayer.com/slide/15197181/92/images/29/Rich+Media+-+Illustrated.jpg. Accessed on September 15, 2021. | 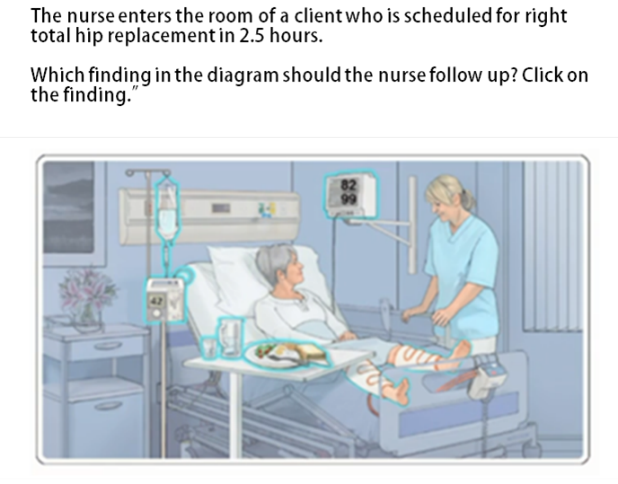 |
|  | multimedia-responsive |  |  |  |
|  | navigation | Dynamic exhibits & constructed response | **Characteristics:** It provides a clickable bar where examinees can browse various clinical information collected with time intervals to demonstrate an understanding of information based on the time flow. It evaluates the ability to resolve practice-focused problems in the clinical field.  **Advantages:** It presents situational problems close to actual clinical settings that change over time in a panoramic format.  **Limitations:** It may take time to solve a question because an examinee may need to understand a clinical situation over the time of the question.  **Reference:** Saunders comprehensive review for the NCLEX- PN® examination (8th ed.). p. 64. E-book. https://yourknowledgedigest.files.wordpress.com/2020/04/saunders-comprehensive-review-for-the-nclex-rnc2ae-examination-8th-edition.pdf. Accessed on January 15, 2022. Step 2. Accessed on January 15, 2022.  Photo credit: https://slidetodoc.com/next-generation-nclex-ngn-overview-phil-dickison-ph/. Accessed on September 15, 2021. | 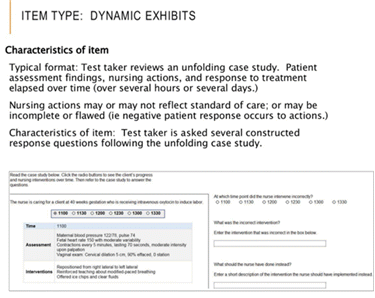 |
|  | chronicle information |  |  |  |
| 25 | Navigation | Dynamic sieving | **Characteristics:** It contains a wide range of information, more than needed, to solve a test question. It measures the ability to select specific information relevant to the situation encountered in recent clinical settings simulating dealing with complex data resources from a drug information website and electronic medical records.  **Advantages:** It can evaluate the ability to select pertinent information among an abundance of information often encountered in clinical fields.  **Limitations:** It requires the collection of question materials. It also needs technical support for security of the relevant data used in the question.  **Reference:** Developed by the study team. Photo credit: https://www.healio.com/cardiology/learn-the-heart/cardiology-review/topic-reviews/atrial-fibrillation. Accessed on September 15, 2021. | 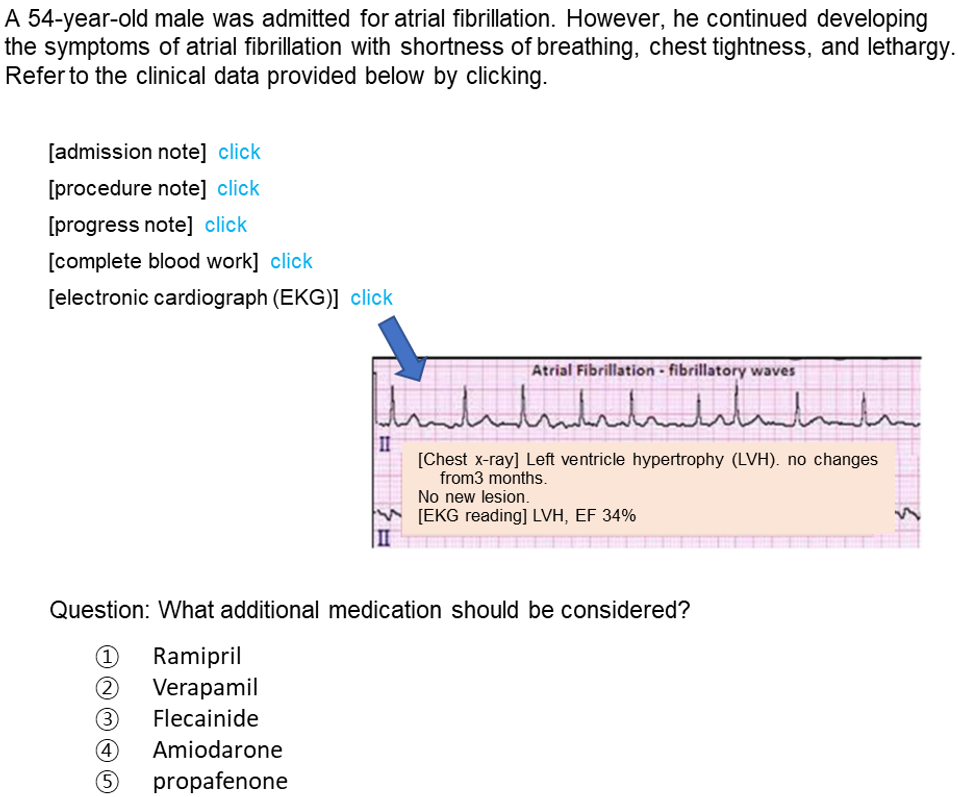 |
|  | mixed information |  |  |  |
| 26 | Simulation-tool | Chat | **Characteristics:** It provides a chat tool and measures the ability of solving problems through conversation and communication over a text chatting.  **Advantages:** It simulates recent healthcare working environments using advanced digital technology with the increase of communication via chat room.  **Limitations:** It needs reactive response technology support to show each response in order.  **Reference:** Developed by the study team. | 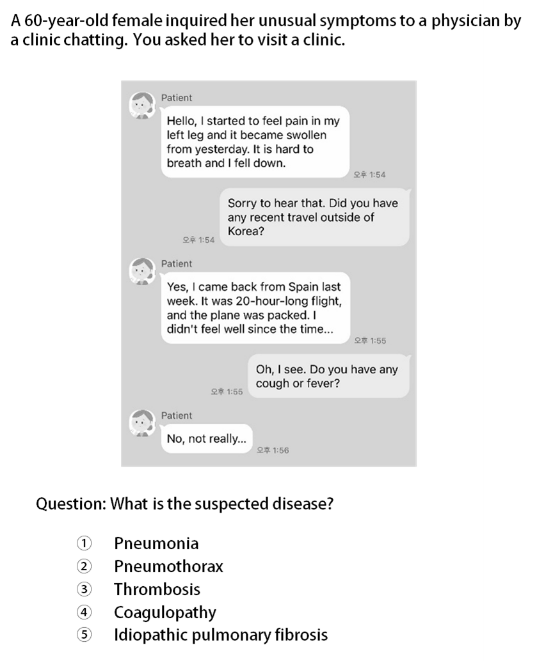 |
|  | chatbot |  |  |  |
| 27 | Simulation-tool | Machine operation_calculator | **Characteristics:** It provides a software application to assess the ability of operating a machine to solve the question.  **Advantages:** It can evaluate the actual ability to calculate using a calculator that often occurs in daily clinical practice. It can limit the possibility of guessing the correct the answer by chance. It measures the ability to calculate biometric indexes, dose, etc. by directly using a calculator.  **Limitations:** It requires inserting a device to operate.  **Reference:** Korea Health Personnel Licensing Examination Institute. Development of CBT-based national licensing examination for health professionals. https://rnd.kuksiwon.or.kr/last/selectFileDown.do?attach_id=2016082400003, p.28, 2014. Accessed on September 15, 2021.  NCLEX Exam. Wendt A, Kenny LE, Marks C. Assessing critical thinking using a talk-aloud protocol. CLEAR Exam Review, 2007;18(1):18-27. Accessed on September 15, 2021.  Photo credit: https://www.ncsbn.org/public-files/Assessing_Critical_Thinking_Talk_Aloud_Protocol.pdf. p.20  https://image.slidesharecdn.com/mapstudentpowerpointpresentation-120904133832-phpapp01/85/map-test-student-presentation-8-320.jpg?cb=1667612047. | 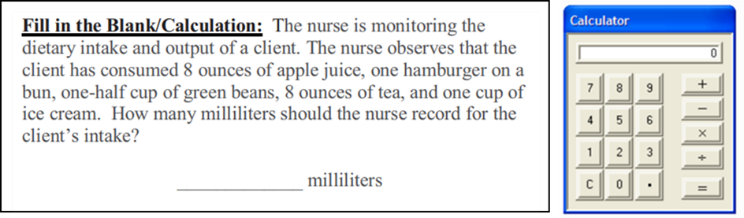 |
|  | calculator |  |  |  |
| 28 | Simulation-tool | Pattern match with JavaScript Molecular Editor (JSME) | **Characteristics:** It uses the interactive editing program JSME based on JavaScript language. It allows editing of molecule structures directly within a web browser using the ‘pattern match algorithm.  **Advantages:** It can evaluate accurate knowledge by having the examinee directly produce the structure or action.  **Limitations:** It may be difficult in scoring and can have a high difficulty level.  **Reference:** Moodle. Pattern match with molecular editor. https://moodle.org/plugins/qtype_pmatchjme. Accessed on January 15, 2022. Photo credit: https://moodle.org/pluginfile.php/50/local_plugins/plugin_screenshots/229/pmatchjme.png?preview=bigthumb. | 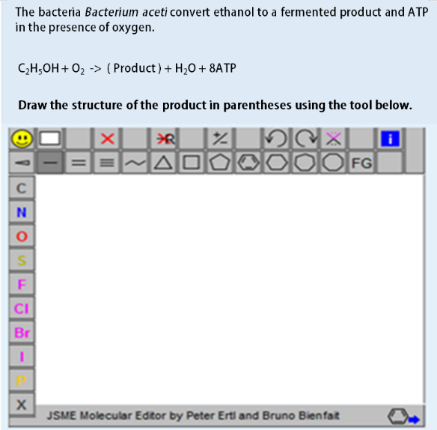 |
|  | drawing agent |  |  |  |
| 29 | Simulation-tool | Searching information | **Characteristics:** It measures a test taker’s ability to acquire information through a database search such as chemicals, medical and drug information, guidelines and regulations.  **Advantages:** It is a highly demanded skill that healthcare professionals need to be familiar with.  **Limitations:** It needs technical support for examinees to access online databases and requires test security.  **Reference:** Developed by the study team. | 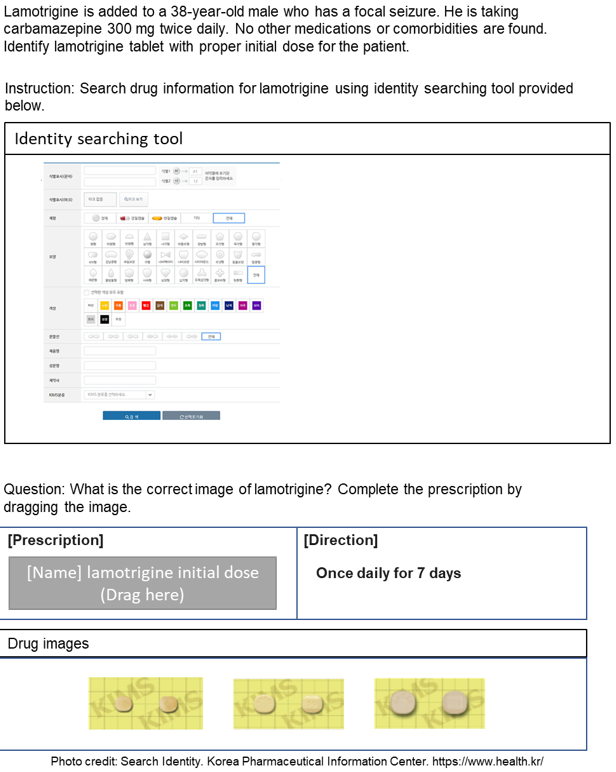 |
|  | database |  |  |  |
| 30 | Spotting on image | Click | **Characteristics:** It involves the action of clicking on a correct answer among several options that may present as forms of texts or images.  **Advantages:** The examinee can intuitively grasp the question and choose the answer from displayed selections through a multi-angled thinking process. If it is set without a limit to the number of options, it may evaluate examinees’ open-ended problem-solving ability. It can be utilized in combination with other item types.  **Limitations:** The technical support to respond to the location of clicks in relation to the answer point. When there is more than one answer, allocation of partial points needs to be discussed.  **Reference:** Developed by the study team.  Photo credit: Encyclopaedia Britannica. Structure of a typical bacterial cell. https://www.britannica.com/science/antibiotic-resistance#/media/1/1027479/129671. Accessed on March 12, 2023. | 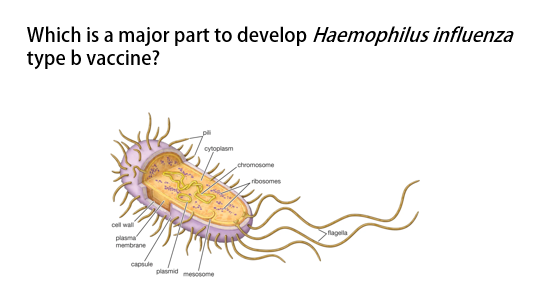 |
|  | structural image |  |  |  |
| 31 | Spotting on image | Hot spot | **Characteristics:** It requires selecting the corresponding response by clicking a correct answer in the presented image, graph, sentence, etc. It is similar with the item type of click, but it does not provide probing marks to show potential options.  **Advantages:** It can measure the ability of understanding an exact anatomical/structural knowledge without probing a label or indicating the boundary of selectable option sites.  **Limitations**: It needs technical support to configure the allowed ranges for clicking for answers and it can increase the level of difficulty.  **Reference:** NCLEX Hot-Spot Questions. https://h5p.org/h5p/embed/85890. Accessed on September 15, 2021.  Photo credit: https://www.chemimpex.com/product/productinfo/imatinib-mesylate/27352?cid=3780. | 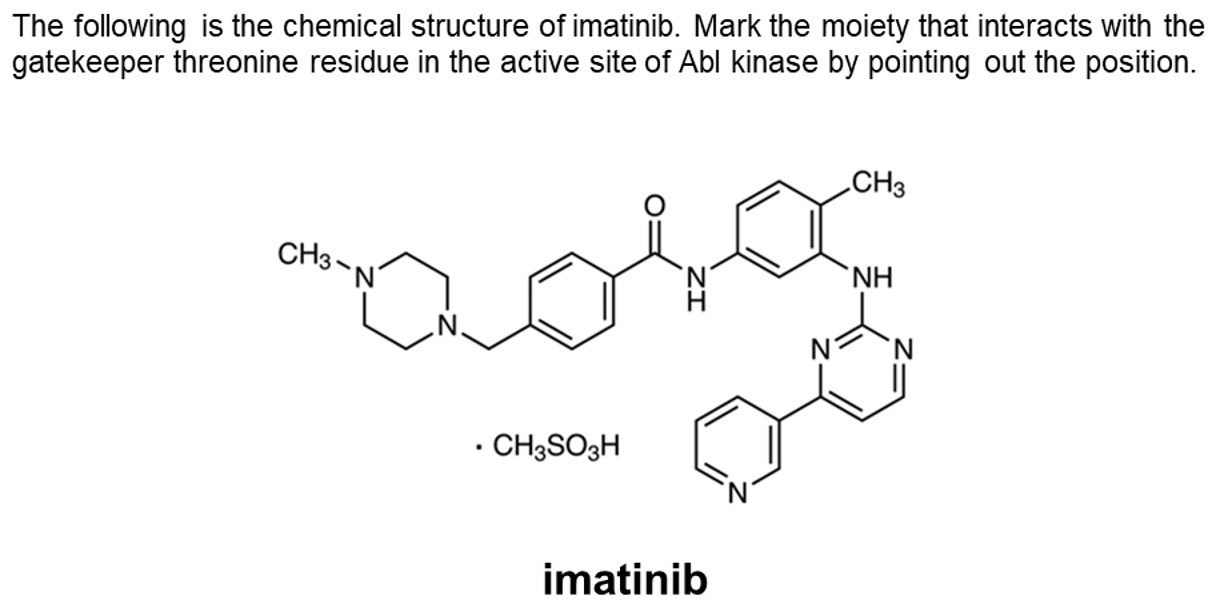 |
|  | structural image |  |  |  |
| 32 | User-responsive | Action button | **Characteristics:** To measure the ability of dynamic action processing (poring, measuring, etc.) in experimental, medication preparation, and treatment procedures (such as airway intubation and wound disinfection). When mixing suspension, an examinee clicks one of the action buttons to evoke the action required to solve a test question.  **Advantages:** It manifests simulation of actual skills that are visually moving by clicking action buttons. The examinees should be able to perform the skills through tests.  **Limitations:** The examinee should be familiar with the structure of the item types.  **Reference:** Developed by the study team.  Photo credit: Korea pharmaceutical information center. amocla syrup® https://www.health.kr/searchDrug/result_drug.asp?drug_cd=2019101800045. Accessed on September 12, 2021. | 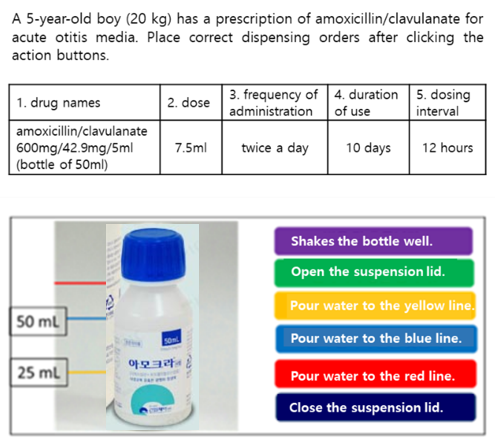 |
|  | button |  |  |  |
| 33 | User-responsive | Peek-a-boo answer | **Characteristics**: It provides the changes of states in the form of images rather than texts to ask processes such as synthesis reactions or treatments. When the image is clicked, the state changes, and it requires examinees to select the correctly changed image as the answer.  **Advantages:** It is suitable to question about reactions, preparation processes, and prognosis of diseases.  **Limitations:** It requires technical support to change specific pictures by clicking each picture.  **Reference:** Developed by the study team. | 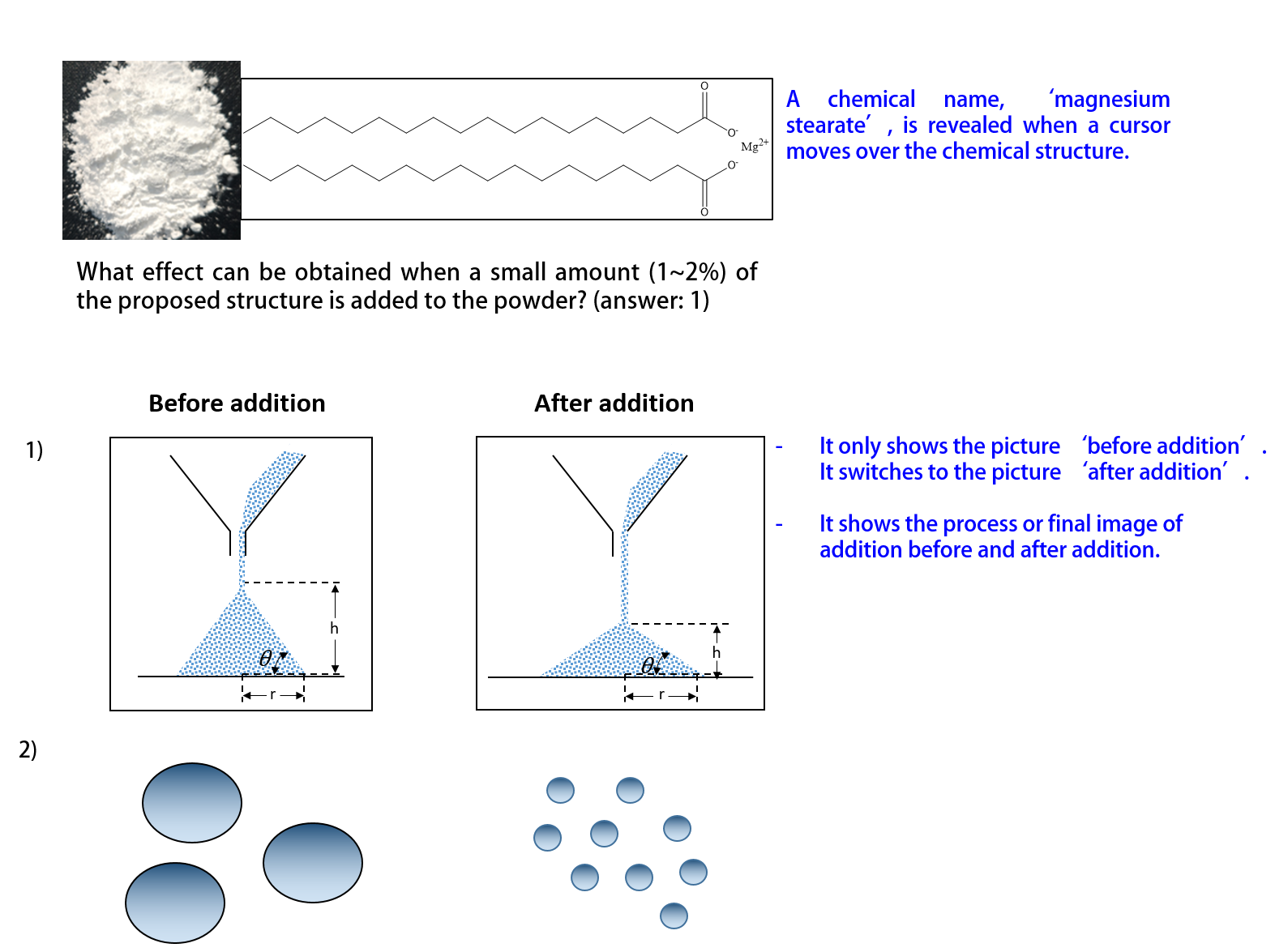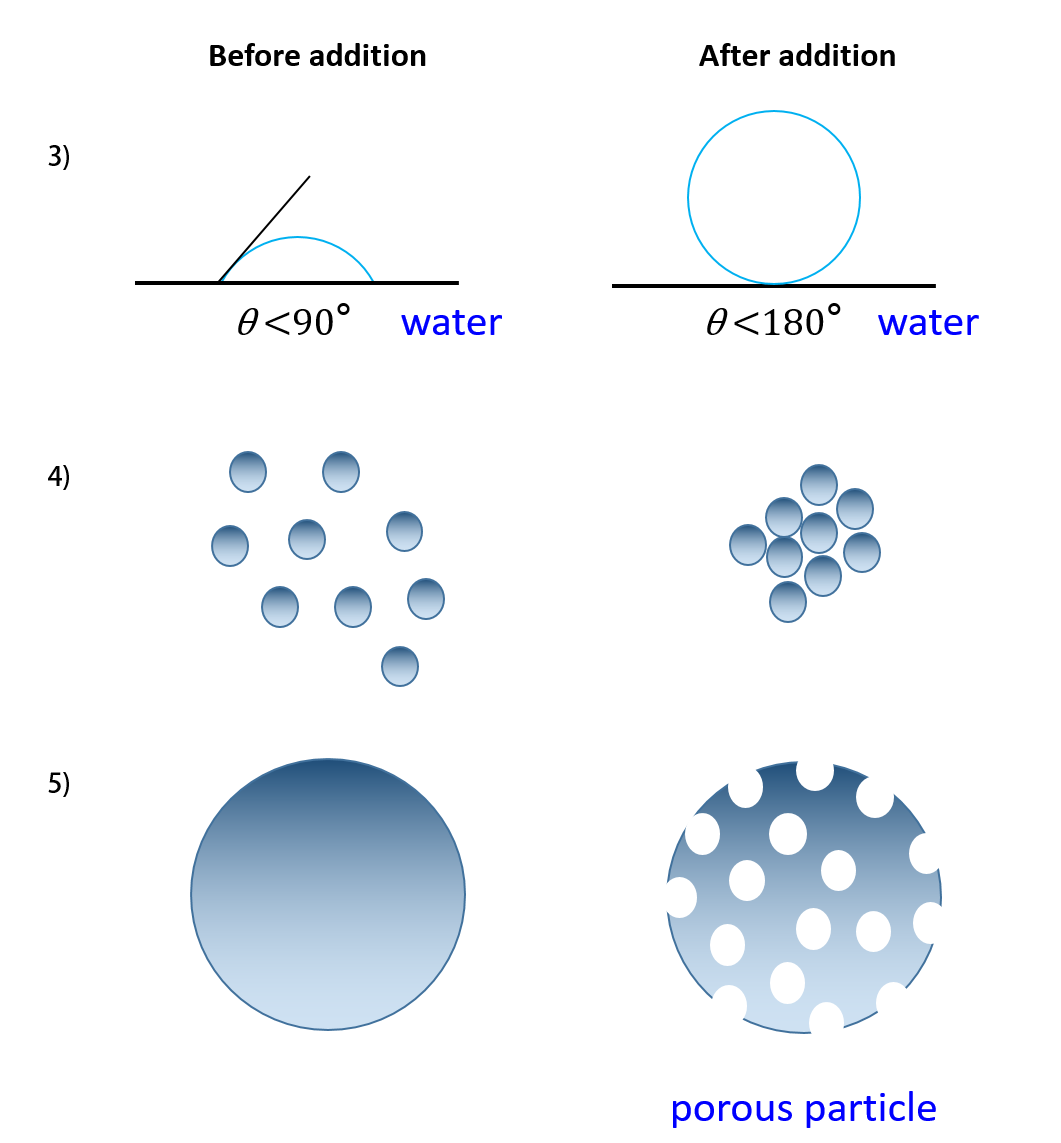 |
|  | reactive image |  |  |  |
| 34 | User-responsive | Trigger | **Characteristics:** It provides auxiliary information to solve a question as a popup when the mouse is placed on the specific part of the text or picture in a test question.  **Advantages:** It allows examinees to obtain clues regarding the definitions of words or descriptions of images that help solve a question.  **Limitations:** It requires technical support for the pop-up function to respond to a cursor.  **Reference:** Developed by the study team. | 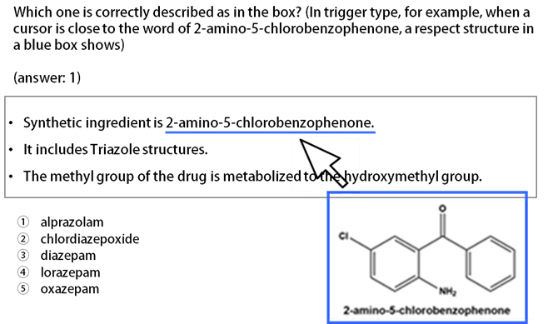 |
|  | pop-up |  |  |  |
| 35 | Miscellaneous | Abstract format | **Characteristics:** It measures the ability of interpreting information presented in an abstract format of an article in a given amount of time. It starts by reading several paragraphs and responding to the following questions based on the content of the written materials in the abstract format.  **Advantages:** It can assess comprehensive knowledge of clinical reasoning using a full abstract from a primary journal or a review summary.  **Limitations:** The length of questions to decrease the time per question needs to be controlled.  **Reference/Photo credit:** United States Medical Licensing Examination (USMLE). Step 2 Clinical Knowledge (CK). https://www.usmle.org/sites/default/files/2021-08/Step_2_CK_Sample_Items.pdf. p.6-7. Accessed on September 15, 2021. | 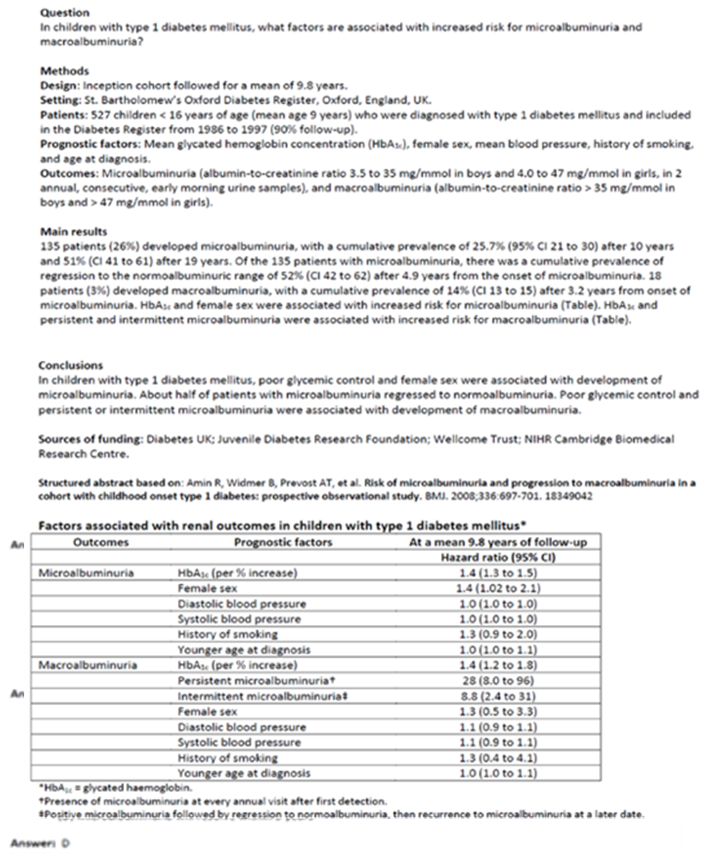 |
|  | abstract reading |  |  |  |
| 36 | Miscellaneous | Calculation | **Characteristics:** It evaluates practical arithmetic calculation abilities and rules out possible guesswork.  **Advantages:** It can evaluate the actual ability of manual calculation which occurs in everyday clinical practice. It excludes the possibility of choosing the correct answer by guessing.  **Limitations:** It may allow for examinees to use blank paper or a note pad app to solve a question. It needs to ensure test time security.  **Reference:** NCLEX Exam. Wendt A, Kenny LE, Marks C. Assessing critical thinking using a talk-aloud protocol. CLEAR Exam Review, 2007;18(1):18-27.  Photo credit: https://www.ncsbn.org/public-files/Assessing_Critical_Thinking_Talk_Aloud_Protocol.pdf. p.20. Accessed on September 15, 2021. | 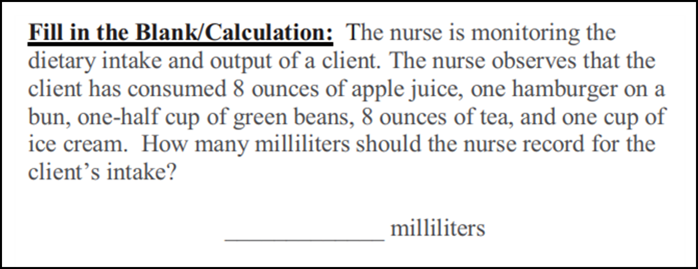 |
|  | short answer |  |  |  |
| 37 | Miscellaneous | Combined | **Characteristics:** It combines at least two different item types.  **Advantages:** It can evaluate the examinee’s ability from various aspects using different item types. Any item types can be used to create a variety of combination types.  **Limitations:** There may be difficulty in operation and technical support for implementing several item types in one question.  **Reference:** Combined question types. Moodle. https://moodle.org/plugins/qtype _combined. Accessed on September 15, 2021.  Photo credit: https://moodle.org/pluginfile.php/50/local_plugins/plugin_description/771/combinedcomplete.png. | 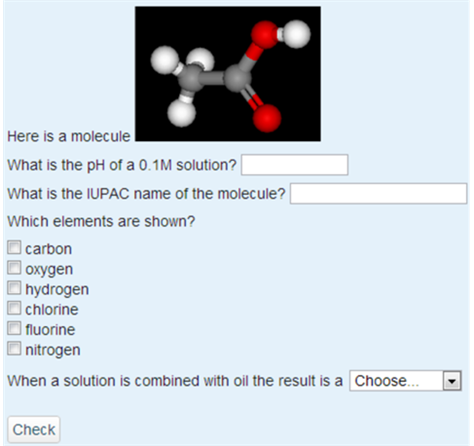 |
|  | mixed type |  |  |  |
| 38 | Miscellaneous | Extended | **Characteristics:** It includes a case description in a stem and provides multiple lead-in questions. It can produce a variety of extended styles of item types, like the item types of extended drag and drop, extended hot spot text, extended matching, extended multiple response, extended ranking, and extended rich media scenario illustrations.  **Advantages:** It can offer various case-contained questions effectively using various item types in the options.  **Limitations:** It may require time to solve the questions, and the level of difficulty needs to be controlled. It should have technical support to implement the item types.  **Reference:** Extended multiple response. https://www.ncsbn.org/public-files/2018_Webinar_NGN.pdf p.19 Accessed on September 15, 2021. |  |
|  | multiple sub-questions |  |  |  |
| 39 | Miscellaneous | Multiple ability assessment | **Characteristics:** It evaluates the ability to use different kinds of skills simultaneously to solve a subject.  **Advantages:** It measures two or more abilities at once via a set of questions.  **Limitations:** Because it may require two or more sequential questions, it may not be appropriate to evaluate one ability at one question.  **Reference:** Korea Health Personnel Licensing Examination Institute. Development of CBT-based national licensing examination for health professionals. https://rnd.kuksiwon.or.kr/last/selectFileDown.do?attach_id=2016082400003, p.28, 2014. Accessed on September 15, 2021.  **Photo credit** NWEA (Northwest Evaluation Association) Math Exemplars. https://www.maine.gov/doe/sites/maine.gov.doe/files/inline-files/Math%20Exemplars%20Grade%207%20with%20Front%20Matter_0.pdf. p.41, 2020. Accessed on September 15, 2021. | 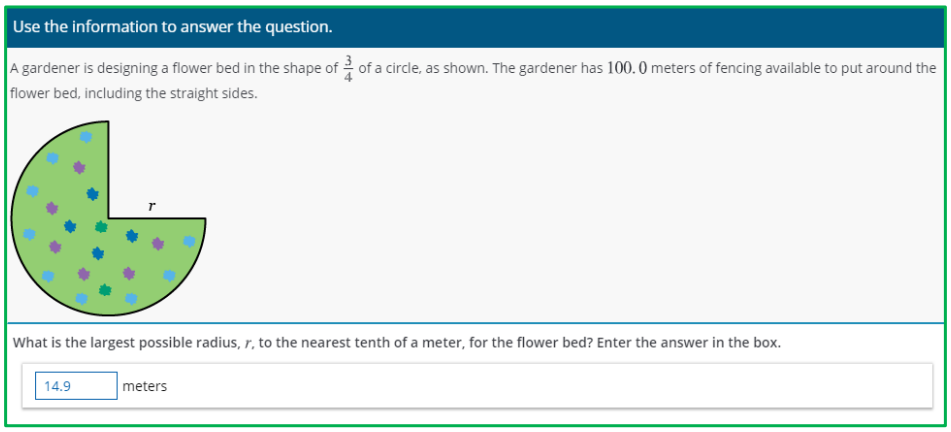 |
|  | multifaceted |  |  |  |
| 40 | Miscellaneous | Order set | **Characteristics:** It asks the questions sequentially. The questions are based on a description of a scenario such as a patient case.  **Advantages:** It asks detailed sub-questions related to the preceding questions.  **Limitations:** The answer to a preceding question may affect the answer to the following question. The question needs to have a sequential structure.  **Reference/photo credit:** United States Medical Licensing Examination (USMLE). Step 2 Clinical Knowledge (CK). https://www.usmle.org/sites/default/files/2021-08/Step_2_CK_Sample_Items.pdf. p. 5. Accessed on September 15, 2021. | 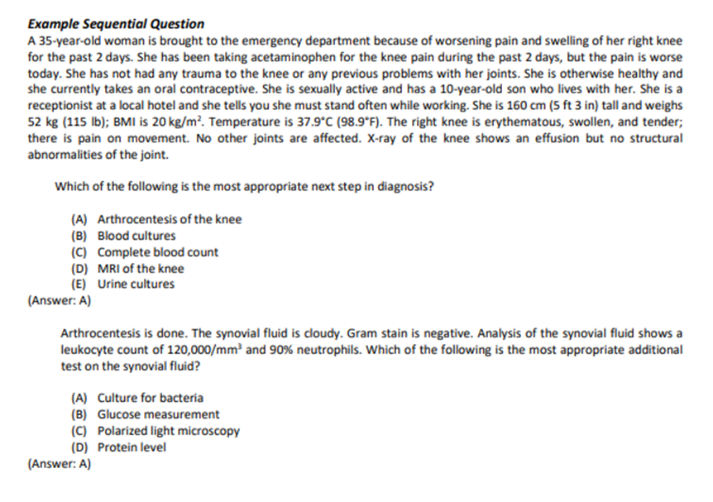 |
|  | sequential question |  |  |  |
| 41 | Miscellaneous | True or false | **Characteristics:** It requires choosing true or false.  **Advantages:** It can evaluate the ability of understanding principals or facts in a simple format.  **Limitations:** It tends to test memorized knowledge. Scoring system should be discussed.  **Reference:** [OX quiz] PEET biology. [Cell membranes and cell membrane transporters]. https://post.naver.com/viewer/postView.nhn?volumeNo=17005997& memberNo=5515487&vType=VERTICAL. Accessed on September 15, 2021.  Photo credit: https://post-phinf.pstatic.net/MjAxODExMDFfMTI5/MDAxNTQxMDU2MDk2ODUy.2ZHfXBfT1DH3eRxkZY6LH57OkvFJb6pqA4YK1-kNGyog.CFrSFi2WBFJFRFX1OZN9aca-exyMmZmU5vlfkGotHb8g.PNG/%EC%95%BD%ED%95%99%EB%8C%80%ED%95%991.png?type=w1200. | 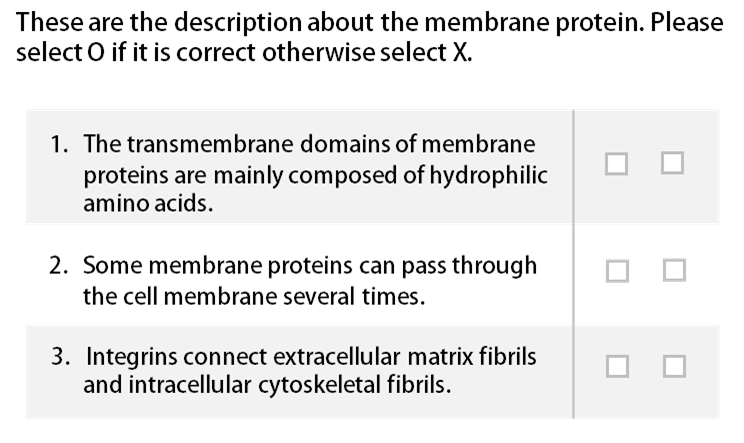 |
|  | two choices |  |  |  |
